# Myelin Membrane Assembly Is Driven by a Phase Transition of Myelin Basic Proteins Into a Cohesive Protein Meshwork

**DOI:** 10.1371/journal.pbio.1001577

**Published:** 2013-06-04

**Authors:** Shweta Aggarwal, Nicolas Snaidero, Gesa Pähler, Steffen Frey, Paula Sánchez, Markus Zweckstetter, Andreas Janshoff, Anja Schneider, Marie-Theres Weil, Iwan A. T. Schaap, Dirk Görlich, Mikael Simons

**Affiliations:** 1 Max Planck Institute of Experimental Medicine, Göttingen, Germany; 2 Department of Neurology, University of Göttingen, Göttingen, Germany; 3 Institute of Physical Chemistry, University of Göttingen, Göttingen, Germany; 4 Max Planck Institute for Biophysical Chemistry, Göttingen, Germany; 5 Third Institute of Physics, University of Göttingen, Göttingen, Germany; 6 Centre for Nanoscale Microscopy and Molecular Physiology of the Brain (CNMPB), Göttingen, Germany; 7 German Center for Neurodegenerative Diseases (DZNE), Göttingen, Germany; Stanford University School of Medicine, United States of America

## Abstract

Rapid conduction of nerve impulses requires coating of axons by myelin. To function as an electrical insulator, myelin is generated as a tightly packed, lipid-rich multilayered membrane sheath. Knowledge about the mechanisms that govern myelin membrane biogenesis is required to understand myelin disassembly as it occurs in diseases such as multiple sclerosis. Here, we show that myelin basic protein drives myelin biogenesis using weak forces arising from its inherent capacity to phase separate. The association of myelin basic protein molecules to the inner leaflet of the membrane bilayer induces a phase transition into a cohesive mesh-like protein network. The formation of this protein network shares features with amyloid fibril formation. The process is driven by phenylalanine-mediated hydrophobic and amyloid-like interactions that provide the molecular basis for protein extrusion and myelin membrane zippering. These findings uncover a physicochemical mechanism of how a cytosolic protein regulates the morphology of a complex membrane architecture. These results provide a key mechanism in myelin membrane biogenesis with implications for disabling demyelinating diseases of the central nervous system.

## Abstract 2

Myelin basic protein undergoes a phase transition from a cytoplasmic soluble pool into a cohesive functional amyloid-like assembly; this may be one mechanism of myelin membrane biogenesis.

## Author Summary

Myelin is a specialized membrane that covers axons and serves as an insulator to enable the fast conduction of the action potentials. The importance of myelin membrane is highlighted in demyelinating diseases such as multiple sclerosis, which lead to severe neurological disability. Here, we describe a physicochemical mechanism of how myelin is generated and assembled. We find that myelin basic protein (MBP) molecules undergo a phase transition into a cohesive meshwork at the membrane interface, which drives structural changes in the membranes. We provide evidence that the interaction of myelin basic proteins with the inner leaflet of the myelin bilayer results in charge neutralization and triggers self-association of the protein into larger polymers. Interactions between MBP molecules are mediated by hydrophobic phenylalanine residues and amyloid-like association. We propose that phase transition of MBP from a cytoplasmic soluble pool into a cohesive functional amyloid-like assembly is one of the key mechanisms in myelin membrane biogenesis.

## Introduction

Compartmentalization and spatial organization of molecules is essential to establish functionally specialized domains within a cell. Segregation of molecules can occur over several length scales ranging from the formation of complexes of few interacting molecules to the generation of micrometer-sized domains. Whereas structural biology has provided us with a wealth of knowledge of how specific molecular interactions occur within macromolecular complexes, little is known about the rules that drive segregation of molecules into large collectives. Phase separations, which constitute a well-recognized phenomenon in nonbiological system, are emerging as a powerful mechanism of how cells organize molecules over larger length scales [1],[2]. One example is the formation of non-membrane-bound organelles within the cytosol [3],[4]. Less is known about how phase separations structure lipid membranes. Here, we addressed this issue using myelin as a model membrane. Myelin is an insulating membrane of vital importance required for the fast conduction of action potential [5]–[9]. It is formed by oligodendrocytes that have the intrinsic capacity to wrap their plasma membrane multiple times around an axon to form a multilayered stack of compacted membranes [10]. The more loosely packed and structurally distinct paranodal loops are localized to the boundaries of compacted myelin [11],[12]. One striking feature of compacted myelin is its unusual molecular composition. Even if myelin is continuous with the plasma membrane, its composition is very different. Around 78% of its dry weight are lipids, whereas only few proteins reside within compacted myelin of which myelin basic protein (MBP) is one of the two most abundant proteins. Its remarkable features include the intrinsically disordered polypeptide chain and the strong basic character with a charge of +20 at physiological pH [13]–[15]. One essential function of MBP is to bring the opposing cytoplasmic surfaces of the myelin membrane closely together [16]. Within the cytoplasmic space MBP forms a size-selective barrier, which prevents the diffusion of most soluble and membrane proteins into the myelin sheath [17]. Here, we address the underlying principles of MBP assembly. We provide evidence that a phase transition into cohesive functional amyloid-like assemblies is one of the key mechanisms in myelin membrane biogenesis in the central nervous system. We propose that this phase transition is essential for protein and cytosol extrusion, and the control of myelin membrane zippering.

## Results

### MBP Drives Protein Extrusion from Membrane Sheets

**Figure 1:**
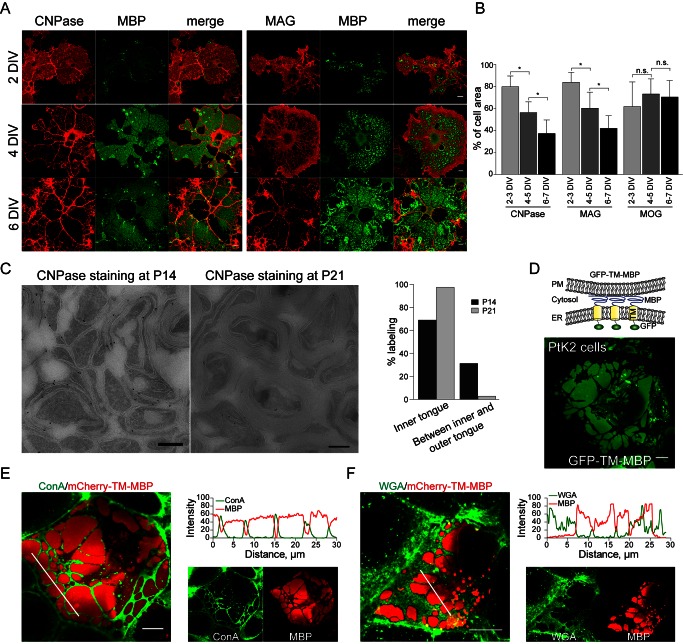
MBP drives protein extrusion. (A) Depletion of CNPase from myelin membrane sheets over time. Scale bar, 10 µm. (B) Quantification of the percentage of cell area occupied by the indicated proteins at different stages of development. MOG, a protein that localizes to the compacted myelin sheets, is shown as a control. Bars show mean ± SD (*n* = 20, *p<0.05, ANOVA). (C) In vivo localization of CNPase in the myelin sheaths of optic nerves at P14 and P21. Scale bar, 500 nm. Quantification for percentage of total labeling of CNPase in inner tongue and between the inner and outer tongue. Bars show mean of 100 myelinated axons. (D) Schematic representation of the ER-PM assay in PtK2 cells. The cytoplasmic domain of an integral membrane protein is replaced by MBP (GFP-TM-MBP). GFP is fused to the N-terminus for visualization. Upon expression in PtK2 cells, the fusion protein forms sheet-like ER-plasma membrane contact sites. (E, F) Visualization of surface glycoproteins using fluorophore-conjugated lectins: concanavalin A (ConA) and wheat WGA in PtK2 cells expressing mCherry-TM-MBP. Scale bar, 10 µm. Most glycoproteins are depleted from the patches as shown by the intensity profile plots for the indicated lectins along the marked lines in the merged images.

We have previously shown that most soluble and membrane proteins are excluded from myelin and MBP plays a role in this exclusion [17]. However, it is unclear how MBP forms such a size-selective barrier. To address this issue, we employed primary cultures of oligodendrocytes that polarise their plasma membrane into large, flat myelin membrane sheets and tubular processes. The molecular composition of myelin membrane sheets is very similar to in vivo compacted myelin [17]. To analyze the segregation of proteins from myelin membrane sheets, we followed the distribution of two proteins that are excluded from compacted myelin: the myelin-associated glycoprotein (MAG), a transmembrane protein, and 2′3′-cyclic nucleotide 3′-phosphodiesterase (CNPase), a peripheral membrane protein. Surprisingly, sheet formation did not immediately trigger protein segregation. First, sheets formed, in which CNPase and MAG were uniformly distributed. Only later, at a time when MBP appeared and accumulated in multiple distinct regions within the sheets, was the exclusion of CNPase and MAG observed (Figure 1A and 1B). Next, we analysed the distribution of CNPase within myelin sheaths by immunoelectron microcopy in cryosections of optic nerves derived from postnatal day 14 (P14) and 21 mice in vivo (Figure 1C). We found that CNPase was distributed homogenously within the myelin lamellae at early stages of myelination, but with the compaction of myelin bilayer at P21, it was mainly restricted to the adaxonal region. These data suggest that proteins are extruded from the myelin sheath and thus raise the question concerning the underlying mechanisms.

### Oligomerization of MBP

**Figure 2:**
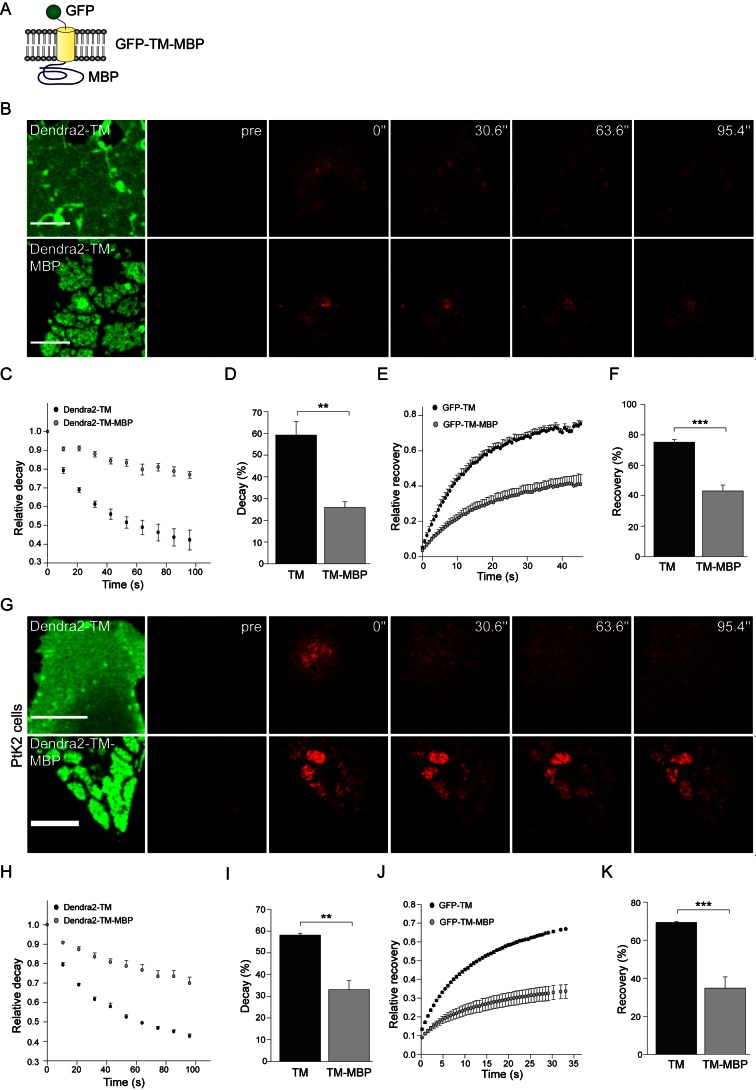
Low mobility of MBP domains. (A) Schematic representation of the reporter construct used to measure mobility of MBP in primary oligodendrocytes. (B) Dendra2 was fused to the N-terminus of either TM (Dendra2-TM) or TM-MBP (Dendra2-TM-MBP) and expressed in primary oligodendrocytes. A squared region of interest was photoconverted from green-to-red via excitation with 405 nm laser. The decay of signal in the photoconveted region of interest was measured over time. Decay of photoconverted signal with time is shown in the representative, zoomed-in images for primary oligodendrocyte cultures expressing either Dendra2-TM or Dendra2-TM-MBP. Scale bar, 10 µm. (C) Curves depict the decay of signal over time for the indicated constructs. (D) Average decay after photoconversion. Bars represent mean ± SEM (*n* = 3, **p<0.01, *t* test). (E) Fluorescence recovery was monitored in primary cells expressing GFP-TM or GFP-TM-MBP after bleaching a squared region of interest. Recovery curves are presented in the form of graphs. (F) Average recovery after photobleaching. Bars represent mean ± SEM (*n* = 3, ***p<0.001, *t* test). (G) Dendra2 was fused to the N-terminus of either TM (Dendra2-TM) or TM-MBP (Dendra2-TM-MBP) and expressed in PtK2 cells. A squared region of interest was photoconverted from green-to-red via excitation with 405 nm laser. The decay of signal in the photoconveted region of interest was measured over time. Decay of photoconverted signal with time is shown in the representative, zoomed-in images for PtK2 cells expressing either Dendra2-TM or Dendra2-TM-MBP. Scale bar, 10 µm. (H) The decay of signal is presented in the form of curves. (I) Average decay after photoconversion. Bars represent mean ± SEM (*n* = 3, **p<0.01, *t* test). (J) Fluorescence recovery was monitored in PtK2 cells expressing GFP-TM or GFP-TM-MBP after bleaching a squared region of interest. Recovery curves are presented in the form of graphs. (K) Average recovery after photobleaching. Bars represent mean ± SEM (*n* = 3, ***p<0.001, *t* test).

One possibility is that upon membrane binding, MBP polymerizes into a protein meshwork thereby squeezing other proteins out of the cytocortex. Indeed using a chemical cross-linking approach, we found that MBP forms large oligomeric complexes in primary oligodendrocytes (Figure S1). To further validate these findings, we used photobleaching and photoconversion experiments to determine the diffusional mobility of MBP molecules. Since soluble MBP lost its function after fusion with a GFP (unpublished data), we designed a chimeric construct (MBP fused to the cytoplasmic domain of an integral membrane protein and containing a GFP in the N-terminus; see scheme in Figure 2A) to be able to perform live experiments. The functionality of the chimeric protein was shown in primary oligodendrocytes prepared from *shiverer* mice lacking MBP, where it rescued the function of wild-type MBP in extruding CNPase from the membrane sheets (Figure S2A and S2B). Since the chimeric protein is transported through the biosynthetic pathway, in contrast to wild-type MBP, it also localizes to the processes and cell body of the oligodendrocyte. Consistent with MBP forming high-order oligomers, the diffusional mobility of the chimeric construct was dramatically decreased when MBP was present at its C-terminus (Figure 2B–F).

### Reconstitution of Protein Extrusion

If a network of cross-linked MBP molecules drives protein extrusion, mutant forms of MBP with impaired ability to self-associate should be nonfunctional. To obtain a rapid screening system, we analyzed whether the function of MBP could be analyzed in PtK2 cells, an epithelial cell line with flat morphology. When we expressed MBP as a chimeric construct (GFP-TM-MBP), micrometer-sized patches of MBP appeared and protein extrusion was induced (Figure 1D–F). Surprisingly, surface staining revealed that GFP-TM-MBP generated these patches from intracellular membrane sites (Figure S3A). Co-labelling with the live-ER stain, ER-Tracker red, showed that GFP-TM-MBP was retained in the ER and formed domains at the ER-plasma membrane interface (Figure S3C). When an ER-retention signal was added to GFP-TM-MBP, patches of similar morphology were generated, confirming that domain formation occurred from the ER (Figure S3D). Domain formation was independent of the choice of the transmembrane domain (Figure S4). Next, we studied protein extrusion, which we could demonstrate in a series of different experiments. First, MAG and several other proteins with large cytoplasmic domains (PLP-GFP, CD9-GFP, CD81-GFP, MOG-GFP, and GFP-MAG) were extruded from the plasma membrane where MBP-patches formed (Figure S5A and S5B). Second and consistent with size-dependent protein extrusion, serial truncations of Tmem10 revealed that a size limit of more than 20 amino acids in the cytoplasmic domain results in exclusion from the patches (Figure S5C). Finally, most glycoproteins were extruded from the surface of the cells in areas where MBP-positive domains appeared as demonstrated by staining with the lectins, concanvalin A (ConA), and wheat germ agglutinin (WGA) (Figure 1E and 1F).

The reversibility of protein displacement was shown by treating cells with ionomycin and sphingosine [18],[19] to antagonize the electrostatic interactions of MBP with the plasma membrane (). Live imaging experiments with photobleaching and photoconversion revealed the cohesive nature of MBP domains as observed in primary oligodendrocytes (Figure 2G–K). In addition, when GFP-TM-MBP was imaged together with WGA, WGA-positive domains were sometimes observed within a MBP-positive domain, but were extruded with time (Figure S6C). Although domains usually remained separated, occasionally domain fusion was observed. Thus, MBP-mediated connection of the ER to the plasma membrane results in protein extrusion and generates micrometer-sized, cohesive, protein-poor domains that resemble compacted myelin in its protein composition.

### Self-Association of MBP Molecules Via Hydrophobic Interactions Is Required for Its Function

**Figure 3:**
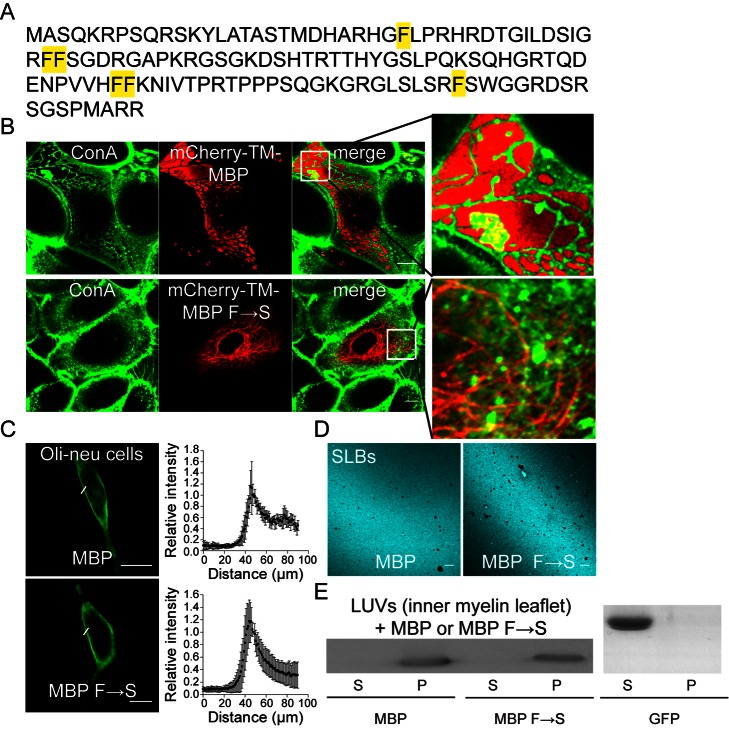
The F→S mutant of MBP loses its ability for protein extrusion, but not for membrane binding. (A) Sequence of the 14 kDa isoform of MBP showing positions of phenylalanine residues that are mutated to serines in the F→S mutant. (B) Distribution of cell surface glycoproteins as visualized by fluorophore-conjugated ConA staining in PtK2 cells expressing mCherry-TM-MBP or mCherry-TM-MBP F→S. Enlarged view of the selected regions in merged images is shown on the right side. (C) Assessment of plasma membrane distribution of soluble MBP and MBP F→S in Oli-neu cells, an oligodendroglial precursor cell line. Scale bar, 10 µm. Relative intensity profile plots along the plasma membrane is shown on the right side (*n* = 20 cells). (D) Typical images of SLBs (with the inner myelin leaflet lipid composition) stained for MBP after incubation with 7 µM of either purified wild-type (MBP) or mutant (MBP F→S). Scale bar, 10 µm. (E) Western blot analysis of supernatant (S) and pellet (P) fractions after incubation of LUVs (large unilamellar vesicles with the inner myelin leaflet lipid composition) with either 3.5 µM of WT MBP, MBP F→S, or recombinant GFP.

Having established a simple assay system, we next probed for mutant forms of MBP with impaired ability to self-associate. Since the analysis of serial truncations mutants of MBP was uninformative (unpublished data), we assumed that the interactions may not be based on simple globular protein modules, but rather depend on multiple weak interactions typical of disordered protein domains. Recently, evidence has been provided that disordered protein domains play a role in phase transitions of proteins into cohesive hydrogels [4]. One prominent example is the nuclear pore proteins that contain FG-repeats, which are natively unfolded and serve as self-association domains [20]. Thus, we created a mCherry-TM fusion construct of MBP, in which the hydrophobic phenylalanine residues had been exchanged to serines. When expressed in PtK2 cells, the protein localized to the ER in tubular structures, but in contrast to the wild-type fusion protein, domain formation was abolished (Figure 3A–B and Figure S7). Similar results were observed when the phenylalanines were exchanged to glycines, but not to the more hydrophobic isoleucines. The effect on domain formation was intermediate with tyrosine substitutions (Figure S8).

One trivial explanation of these results could be the inability of these mutants to bind to the cytoplasmic leaflet of the plasma membrane. This possibility was ruled out by a number of independent approaches (Figure 3C–E). First, when wild-type and F→S MBP were expressed in cells, both proteins were found at the plasma membrane to a similar extent (Figure 3C). Second, purified recombinant F→S and wild-type MBP protein molecules bound to supported-lipid bilayers (with the inner myelin lipid composition) uniformly and could not be washed away as revealed by antibody staining against MBP molecules (Figure 3D). Third, both proteins were able to pull-down liposomes (with inner myelin lipid composition) to the same extent, in contrast to recombinant GFP used as a control protein (Figure 3E).

**Figure 4:**
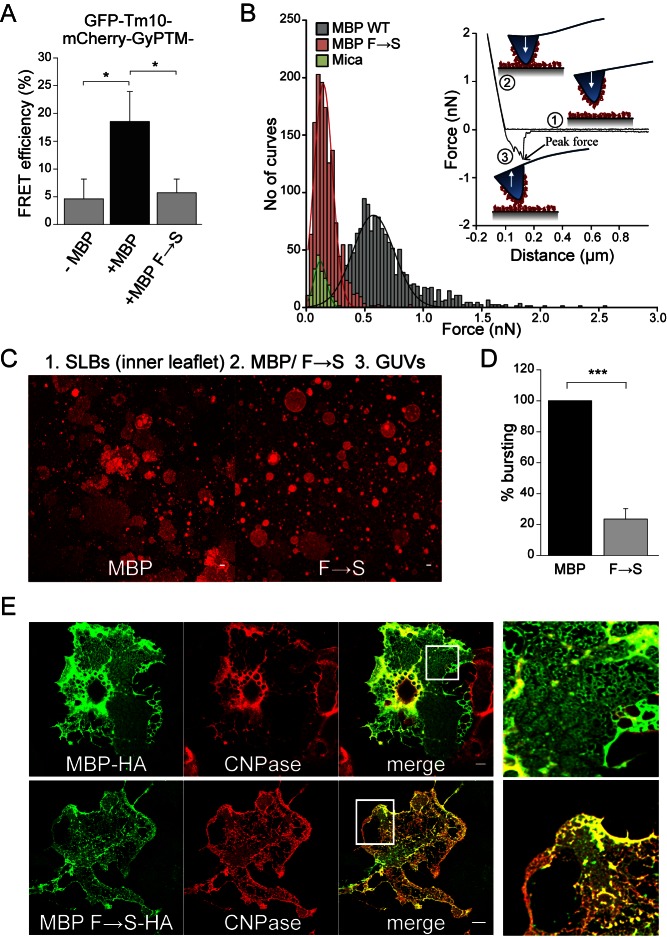
Self-association of MBP molecules via hydrophobic interactions is required for its function. (A) Quantification of FRET efficiency in PtK2 cells expressing GFP-Tm10 (Donor) and mCherry-GyPTM (Acceptor) both harboring at the C-terminal end either wild-type MBP or MBP F→S. While Tm10 represents the transmembrane domain of Tmem10, GyPTM represents the mutated monomeric transmembrane domain of the glycophorin protein. Bars indicate mean ± SD (*n* = 20 cells, *p<0.05, ANOVA). (B) Comparison of interaction forces between wild-type MBP or F→S mutant molecules pre-adsorbed, both to the mica surface and AFM tip. Inset shows the schematic depiction of shape of the curve as cantilever tip approaches the sample surface (1), as tip touches the surface (2), and as tip is retracted from the sample surface (3). Histogram of peak force measured for MBP (black), MBP F→S (red), and buffer (green). (C) Representative images of a biomimetic assay in which MBP or MBP F→S is sandwiched between SLBs (inner myelin leaflet lipid composition) and GUVs (PC∶PS in 3∶1 mole%). Scale bar, 10 µm. (D) Quantification of percentage GUV bursting. Bars show mean ± SEM (*n* = 3 experiments, ***p<0.001, *t* test). (E) *Shiverer* cells at 0 DIV were infected with AAV2 viral particles expressing either wild-type MBP (MBP-HA) or F→S mutant (MBP F→S-HA) containing a C-terminal HA-tag. At 6 DIV, cells were immunostained for CNPase and the HA tags. Expression of MBP-HA induces the depletion of CNPase from regions within the sheets, whereas the F→S mutant fails in extruding CNPase despite entering the sheets of *shiverer* cells. Enlarged view of the selected regions in merged images is shown on the right side. Scale bar, 10 µm.

We next examined the ability of the mutant MBP molecules to self-associate. A FRET assay using GFP-Tm10-MBP as a donor and mCherry-GypTM-MBP (Tm10 represents transmembrane domain of Tmem10 and GyPTM represents monomeric transmembrane domain derived from the glycophorin) as an acceptor revealed much higher FRET ratios between the wild-type MBP fusion proteins in comparison to the F→S mutant (Figure 4A). In addition, we measured interaction forces between MBP molecules located on a mica surface and the tip of a cantilever using atomic force microscopy as described previously [21]. The force versus distance measurements revealed dramatic differences when the F→S mutant was compared to wild-type MBP, in agreement with our conclusion that mutant MBP had lost its ability to self-associate (Figure 4B). The functional consequences of impaired self-association were studied by a number of different assays. First, we employed a biomimetic model system, which examines the interaction of giant unilamellar vesicles (GUVs) to supported lipid bilayers (SLBs) coated with MBP. In this system, MBP is sandwiched between a SLB and GUVs where it induces the spreading of GUVs onto the SLB. When the F→S mutant was analyzed, we observed that the mutant promoted the tethering of GUVs to the SLBs, but it was significantly less efficient in inducing the bursting and spreading of GUVs (Figure 4C and 4D). Next, we expressed soluble MBP and the F→S mutant in *shiverer* mice in vivo and in oligodendrocytes prepared from *shiverer* mice in vitro by using recombinant AAV vectors carrying the MBP promoter as an expression system [22]. We observed that myelin in *shiverer* mice injected with recombinant AAV-wild-type MBP virus was partially compacted and with slightly more wraps as compared to AAV- F→S mutant MBP injected animals (Figure S9). In cell culture we could show that both wild-type MBP and the F→S mutant entered into the membrane sheets of *shiverer* cells, but only the wild-type MBP was able to extrude CNPase (Figure 4E). When we compared the targeting of MBP and the F→S mutant into myelin membrane sheets of wild-type cells, we found that only wild-type MBP localized to the mature myelin membrane sheets of cultured oligodendrocytes (Figure S10A). Thus, it appears that phenylalanine-mediated hydrophobic interactions are not only important for the function of MBP, but also for the targeting into MBP-positive domains. To challenge this conclusion, we tested whether MBP and the F→S mutant were able to route proteins with large cytoplasmic domains piggyback into MBP-positive domains in PtK2 cells. The integral membrane protein Tm10C50 with a 50 amino acid large cytoplasmic domain, which prevents membrane sheet localization, was used as a reporter. Indeed, only when wild-type MBP was fused to the C-terminus of Tm10C50 was the size barrier overcome and localization to MBP-positive domains triggered (Figure S10B). Thus, intermolecular MBP-mediated interactions may drive proteins into the domains. This conclusion was further substantiated using a rapamycin-inducible molecular bridging approach [23]. Using this system, it was possible to immobilize and bridge mCherry into MBP-positive ER-plasma membrane patches (Figure S10C). Taken together, our results suggest that hydrophobic phenylalanine-mediated interactions are required to form a cohesive mesh-like network of MBP molecules between the cytoplasmic leaflets of two opposing membranes. Small proteins appear to diffuse freely through the pores, whereas large proteins are extruded unless they are bound to the meshwork.

### Phase Transition of MBP

**Figure 5:**
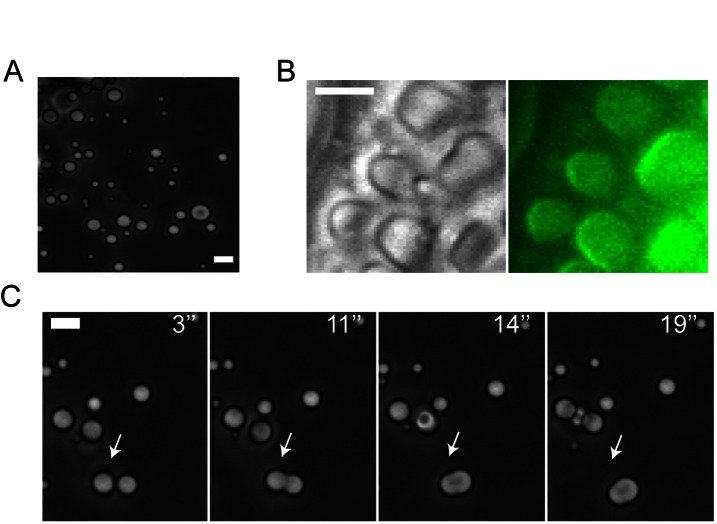
Phase transition of wild-type, but not the F→S mutant of MBP. (A) In basic solution MBP (5 mg/mL) forms droplets as visualized by phase contrast microscopy. (B) Droplets contain Atto-488-labeled MBP (5 mg/mL) as visualized by wide field (right panel) microscopy. (C) Time-lapse images of two merging droplets. Scale bar, 5 µm.

One possible mechanism to explain how MBP functions in forming a semipermeable interface would be through a phase separation. Hence, we analyzed whether phase transition of purified MBP molecules could possibly be triggered in solution. For this, a pH shift was used as a strategy to mimic the in vivo charge neutralization by the negatively charged membranes. Strikingly, beyond a critical concentration, MBP formed a cloudy solution in 20 mM NaOH. Mixing resulted in the fragmentation of initially bicontinuous interconnected phases into µm-sized droplets (Figure 5A). Atto-488–labelled MBP revealed enrichment of the protein in droplets (Figure 5B). Gradually, coalescence of droplets into larger droplets was observed (Figure 5C). Together, this behaviour is consistent with a demixing phase transition in fluid. The phase transition was reversible as the droplets rapidly dissolved upon pH neutralization. Importantly, the solution remained clear and no droplets were formed when similar experiments were performed with the F→S mutant (Figure S11). These results show that MBP possesses the inherent capacity for phase transitions, a process which is critically dependent on hydrophobic phenylalanine-mediated interactions. This surprising observation is in line with recent findings showing phase transitions of soluble proteins in the cytosol that are involved in germline P granule biogenesis, assembly of signalling complexes in the cytocortex, or of cohesive FG domains forming the permeability barrier of nuclear pores [3],[20],[24].

### Amyloid-Like Assembly of MBP

**Figure 6:**
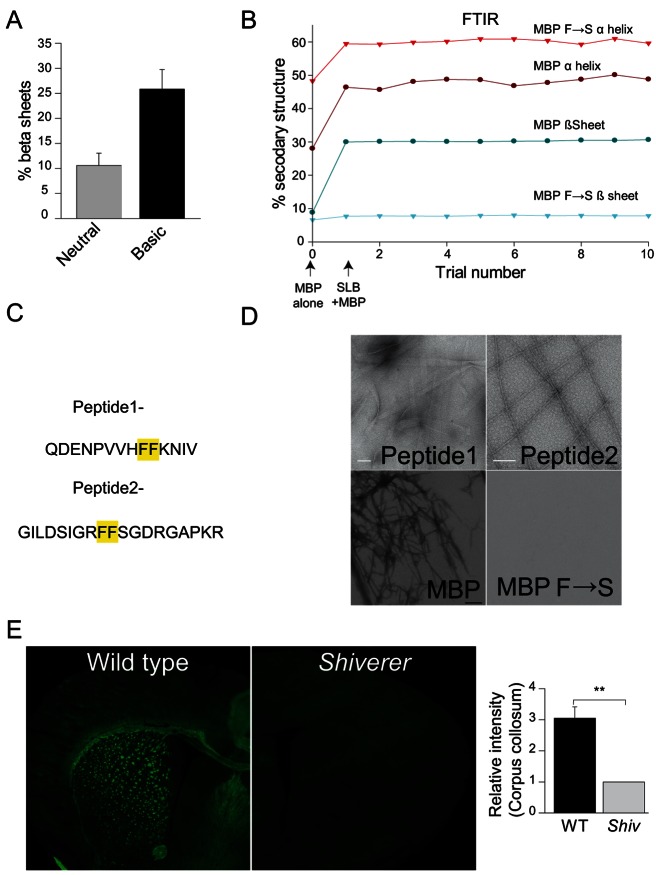
β-sheet structure mediates amyloid-like self-assembly of MBP. (A) Secondary structure determination from FTIR spectroscopy of wild-type MBP in the presence of 20 mM NaOH. Bars represent range from two independent experiments. (B) Secondary structure determination of wild-type and the F→S mutant after addition to the SLBs with the inner myelin leaflet lipid composition. (C) Aggregation-prone stretches within MBP sequence. (D) Transmission electron microscopy of peptide 1 and peptide 2 (left and middle panel) incubated in 25 mM HEPES (pH 7.5), 150 mM KCl, and 0.5 mM MgCl_2_ for several days. The lower panel shows the fibrillar aggregates obtained in 20 mM HCl, 500 mM Na_2_SO_4_, and 5% ethanol for wild-type MBP, but not the MBP F→S mutant. (E) Thioflavin S staining of P18 MBP deficient *shiverer* and wild-type mice brain. Quantification of relative fluorescent intensity in corpus collosum. Bars show mean ± SEM (*n* = 3 animals, **p<0.01, *t* test).

To analyze the underlying secondary structural changes occurring after the phase transition, we used attenuated total reflection fourier transform infrared spectroscopy (ATR-FTIR). An increase in β-sheet structure of purified MBP was observed at basic pH, suggesting its involvement in self-assembly (Figure 6A). Next, we compared the structural changes in wild-type MBP and the F→S mutant upon membrane interaction. As previously shown, MBP in solution displayed very low proportion of ordered secondary structure, but upon addition to the SLBs, the amount of α-helices and β-sheets increased immediately. In a striking contrast to the wild-type MBP, the F→S mutant exhibited α-helical, but only very little β-sheet structure in solution and, importantly, no increase in β-sheet structure was observed after the addition to the SLBs (Figure 6B). Computer algorithms that predict aggregation-prone regions in unfolded protein chains [25] revealed two stretches within the MBP sequence that have a high β-aggregation propensity. Interestingly, both these stretches harbour a pair of phenylalanine residues that were replaced with serines in the F→S mutant (Figure 6C). β-sheet structures are not only a key feature of irreversible protein aggregates seen in the form of amyloid fibres in neurodegenerative diseases, but also of functional and reversible amyloid-like protein interactions sometimes found in normal tissue [26]. We thus speculated that an amyloid-like aggregation process may underlie the self-assembly of MBP. We designed peptides containing the two aggregation-prone regions of MBP. Indeed, after a few days of incubation at 37°C, both peptides assembled into twisted-sheet-like and straight fibres as shown by electron microscopy (Figure 6D). Similar results were obtained with full-length wild-type, but not the F→S mutant of MBP, when incubated for extended times (up to 3 wk) at 37°C under fibrillating conditions (Figure 6D). Previously, fluorescent dyes, which stain amyloids, have been shown to label the white matter in the sections of adult mice brain [27]. We used the amyloid dyes thioflavin S, K114, and BTA1 to image brain sections of P18 wild-type and MBP deficient *shiverer* mice. We chose an early stage of development to ensure that the myelin content was in a similar range in wild-type and mutant animals. While robust staining was observed with the compound in the corpus callosum of wild-type brains, for *shiverer* brains the staining was negligible, suggesting that the dyes indeed detect MBP in vivo (Figure 6E and Figure S12).

## Discussion

Myelin is an essential and highly abundant membrane of the vertebrate brain. To obtain its insulting properties, most soluble and membrane proteins have to be depleted to generate a lipid-rich sheath. We find that one underlying mechanism is the phase transition of MBP molecules into a condensed network that drive protein segregation from the cytoplasmic leaflets of two opposing membranes. Hydrophobic phenylalanine-mediated interactions were required for the formation of the mesh-like network of MBP molecules. Small molecules appear to diffuse freely through the pores of the protein network, whereas larger proteins are extruded unless they are bound to MBP.

What are the molecular properties that define such transitions? Whereas molecular interactions in macromolecular complexes depend on specific modular protein interfaces, which are often sensitive to singe point mutations, phase transition appears to follow different rules [28]. One important feature appears to be the presence of multivalent interaction domains, which often contain low complexity sequences, a typical attribute of many intrinsically disordered proteins [4],[24]. Thus, single point mutations are unlikely to disrupt the phase transition of proteins. A characteristic signature of some proteins undergoing phase separations are repeats of disordered protein domains containing the hydrophobic amino acid phenylalanine and the flexible amino acid glycine [29]. Consistent with this general conceptual framework, we find that MBP loses its intrinsic capacity for phase transitions after multiple phenylalanine mutations.

Strikingly, several intrinsically disordered proteins have been implicated in the formation of pathological amyloid formation in neurodegenerative diseases [30]. Recently, evidence has been provided that many intrinsically disorder proteins with low complexity sequences have the capacity to form amyloid fibrils in phase-separating hydrogels [4]. Here, we show that self-assembly of MBP may also share some features with amyloid-like fibril formation. However, unlike classical pathological amyloid fibrils, which form extremely stable and rigid structures, amyloid-like interactions in physiological assemblies have weaker and reversible connections [26],[31]. Such dynamic amyloid-like interactions have also been described for the permeability barrier of the nuclear pore [32] and RNA granules [4]. Amyloid-like interactions do not depend on specific motifs, but are often seen in structurally disordered protein domains and can even occur between single phenylalanine amino acids [33]. Another interesting example of reversible functional amyloid assemblies is the self-association of hormones in amyloid-like aggregates—a phase transition that is regulated by the low pH inside a vesicle [34]. Thus, the control of a phase transition represents a powerful mechanism to generate sharp transitions between physically and functionally distinct states of a pool of molecules. Here, we propose that the binding of MBP to two membrane surfaces triggers a phase-transition. The interaction to the negatively charged membrane may neutralize the positive charge in MBP and induce self-assembly by loss of electrostatic repulsion. Since phase transitions require critical concentrations of molecules, control of MBP expression level, which has recently been shown to depend on neurons, is likely to be relevant [35]–[37]. Thus, a phase transition of MBP might be coupled to neuronal activity in order to coordinate myelin assembly.

It is interesting to compare the protein meshwork formed by MBP with the permeability barrier of the nuclear pore. FG-repeats of the nuclear pore proteins mediate the assembly of a dense protein meshwork forming a hydrogel-based barrier for nucleocytoplasmic transport [20],[38]. Whereas small proteins diffuse freely through the pores of the barrier, larger proteins require the interaction with receptors to open and enter the meshwork. The barrier formed by MBP may also behave like a selective phase. We found that proteins with large cytoplasmic domains were excluded from protein extrusion when bound to the MBP meshwork. It is conceivable that tight interactions with MBP result in the trapping of proteins within the mesh, whereas proteins that do not interact are forced out of the myelin sheets. In the future it will be interesting to determine the precise structural and energetic requirements that determine whether proteins are recruited into, move through, or are expelled from the meshwork of MBP molecules.

How do phase transitions contribute to the spatial organization of a cell? One example is the generation of non-membrane-bound compartments within the cytosol. Another possibility is that phase transition structure lipid membranes. Indeed, we are able to show that the phase transition of MBP alters membrane composition by propelling protein extrusion. At the same time, the formation of the condensed MBP fluid phase could drive a phase separation in the myelin membrane bilayer. By this means a phase transition in the cytosol may trigger a rearrangement and a condensation of membrane lipids in the forming myelin sheath. A phase transition of proteins impelling a segregation of membrane lipid is an attractive concept that can now be tested with the experimental system established in this study.

In addition, our work may help to understand the mechanism of myelin disassembly in vascular or autoimmune diseases such as multiple sclerosis. A possibility that needs to be addressed in the future is whether a phase transition in the reverse direction induces breakdown and degradation of myelin in demyelinating diseases.

In summary, our findings provide a physicochemical mechanism of how protein self-assembly can provide the forces for long-range segregations within the cytosol at the membrane interface. Such sharp phase transitions might be a general phenomenon of how cells employ physical principles for the compartmentalization of the cytosol ranging from the nuclear pore to germline P granule and the assembly of signalling complexes in the cytocortex [1]–[4],[20],[24],[32].

## Materials and Methods

### Ethics Statement

All animal treatments were approved in advance by the Lower Saxony state authorities (“Niedersächsisches Landesamt für Verbraucherschutz und Lebensmittelsicherheit”; Postfach 39 49; 26029 Oldenburg) for animal experimentation and conducted in accordance with animal protection laws approved by the Government of Lower Saxony, Germany. The Approval ID is 33.7-42502-04-12/0778 and the project name “Mechanismen der chronischen Progredienz bei Multipler Sklerose-3”.

### Antibodies, Plasmids, and Other Reagents

The following antibodies were used in the study: mouse monoclonal IgG antibodies against MAG and MOG (Millipore, Billerica, MA, USA), mouse monoclonal IgG antibodies against MBP (Sternberger, Lutherville, MD, USA), mouse monoclonal IgG antibodies and rabbit polyclonal against myc tag and CNPase (Sigma-Aldrich, Munich, Germany), rabbit polyclonal anti-MBP antibodies (DakoCytomat., Carpinteria, CA, USA), and rabbit polyclonal anti-HA antibodies (Abcam, Cambridge, UK). Secondary antibodies, fluorophore- or peroxidise-conjugated, were purchased from Dianova (Hamburg, Germany). Concanavalin A coupled to either Alexa-594 or Alexa-488 and WGA coupled to Alexa-488 or Tetramethylrhodamine were bought from Invitrogen (Munich, Germany). Ionomycin was purchased from Calbiochem (Darmstadt, Germany), rapamycin from Sigma-Aldrich (Munich, Germany), and sphingosine from Avanti Polar Lipids (Alabaster, AL, USA). 1,2-dioleoyl-*sn*-glycero-3-phosphoethanolamine (PE), porcine brain L-α-phosphatidylinositol-4,5-bisphosphate (PIP2), egg L-α-phosphatidylcholine (PC), porcine brain sulfatide, and porcine brain L-α-phosphatidylserine (PS) were obtained from Avanti Polar Lipids as chloroform stocks. Cholesterol and chicken egg yolk sphingomyelin (eSM) were obtained from Sigma-Aldrich (Munich, Germany). LissamineTM rhodamine B 1,2-dihexadecanoyl-*sn*-glycero-3-phosphoethanolamine (rhodamine-DHPE), Texas Red DHPE, and ER-Tracker Red were from Invitrogen (Munich, Germany). Trans-1-bromo-2,5-bis(4-hydroxystyryl)benzene (K114) and 2-(4″-(methylamino)phenyl)benzothiazole (BTA1) were from Sigma-Aldrich (Munich, Germany) [39].

### Mice, Primary Cell Culture, Cell Lines, Transfections, and Treatments

*Shiverer* mice were maintained on a C57/N background. Genotype of offsprings was determined by PCR. Primary cultures of mouse oligodendrocytes were prepared from newborn P0 NMRI mice as described before [40]. Oligodendrocyte progenitors were shaken off from the bottom layer of astrocytes and plated in Super-Sato medium (DMEM with high glucose supplemented with 1× B27 supplement, 1× B-27 supplement, 2 mM GlutaMAX, 1 mM sodium pyruvate, 1% HS, 50 units/ml each of penicillin and streptomycin, 0.5 mM triiodothyronine, and 0.52 mM L-thyroxine). Transient transfections in primary oligodendrocytes were performed using Lipofectamine 2000 transfection reagent (Invitrogen GmbH, Darmstadt, Germany) according to the manufacturer's protocol. PtK2 cells were kindly provided by C. Eggeling, Max Planck Institute for Biophysical Chemistry. Cells were cultured as described previously in DMEM with the following additions: 10% FCS, 4 mM glutamax, 1 mM pyruvate, and 50 units/ml, each of penicillin and streptomycin. For cell splitting, 0.05% trypsin-EDTA was used. Oli-neu cells were cultured in DMEM with the following additions: 2 mM glutamax, 1 mM sodium pyruvate, 5% horse serum, 1× insulin-selenium-A supplement, 100 µM putrescine dihydrochloride, 0.5 µM L-thyroxine, 0.2 µM progesterone, and 50 units/ml, each of penicillin and streptomycin. For cell seeding, precoated petri dishes or glass coverslips were used. Transient transfections in PtK2 and Oli-neu cells were performed using TransIT-LT1 transfection reagent (Mirus) according to the manufacturer's instructions.

### AAV2 Generation and Injection

Recombinant AAV vectors were propagated in HEK 293 cells by calcium phosphate-mediated co-transfection of the vector plasmid with the helper plasmid pDG (Plasmid Factory). The cells were harvested at 48 h after transfection, and the AAV2 virus particles were purified from cell lysates by iodixanol step gradient centrifugation. Virus was further purified and concentrated by fast-protein liquid chromatography on Heparin affinity columns (Amersham) and titer determined as described [41]. Eluted virus was dialyzed against PBS and stored in single use aliquots at −80°C. Twenty-one-week-old shiverer mice were injected with recombinant AAV2 carrying the MBP promoter as described in [22]. Animals were anesthetized with a ketamine/xylazine mixture before stereotactic injection of 1.5 µl virus suspension (6×10^8^ transducing units/µl) into the corus callosum (coordinates a/p −0.8, m/l −0.6, d/v −1.5 from Bregma) using a glass microcapillar and a motorized injection pump (World Precision Instruments) at a constant flow rate of 250 nl/min. Monastral blue (Sigma-Aldrich) was included in the injections as a marker dye to localize the injection sites. The needle was kept in place for additional 5 min after injection to prevent reflux. Mice were sacrificed 14 d after injection and perfused with paraformaldehyde. All animal procedures were approved by the local animal care committee.

### Immunofluorescence, Microscopy, and Analysis

Immunocytochemistry was performed as described previously [6]. Fluorescence images were acquired on the following set-ups: Leica TCS SP2 AOBS confocal microscope (Leica Microsystems, Mannheim, Germany) equipped with 40× NA 1.25 and 63× NA 1.4 oil plan-apochromat objectives, Carl Zeiss LSM 510 META confocal setup equipped with 63× oil objective, and Leica DMI 6000 fluorescence microscope with adaptive focus control and equipped with 40× NA 1.10 oil plan-apochromat objective. At least three independent experiments were performed for each analysis. Images were processed and analyzed with the public domain Java-based image processing software ImageJ (created by W. S. Rasband, National Institutes of Health, Bethesda, Maryland, USA). Colocalization of the fluorescent signals was estimated by calculating Pearson's correlation coefficient with Intensity Correlation Analysis ImageJ plugin. Quantification of relative cell surface area occupied by the specific proteins was performed with ImageJ using the application: threshold from background, followed by manually defining the area, and finally measuring the area fraction above the threshold. For generating intensity profile plots along the manually defined horizontal line, images were first corrected for the background followed by measurement of average intensity along the length.

### FRAP

For fluorescence recovery after photobleaching (FRAP), cells were transfected with the plasmid of interest. Photobleaching was performed in five scans with the 488/561 laser at full power within 5 µm×5 µm region of interest (zoom-in mode). Pre- and postbleach fluorescence intensities (2 and 20 scans, respectively) were monitored with a laser power of 6% for 488 and 25% for 561. Eight-bit images were recorded every 0.657 s at a resolution of 512×512 pixels, with a scanner speed of 1,000 Hz. Processing and analysis of FRAP data was performed as described [42].

### FDAP

For fluorescence decay after photoconversion (FDAP), cells were transfected with proteins of interest fused to photoconvertible Dendra2. A 5 µm×5 µm region of interest (ROI) was excited with 80% 405 laser for 3–5 cycles in order to photoconvert Dendra2 from green to red. After photoconversion, decay of signal in the ROI was monitored over time using 15% of 561 laser for 500 s with image acquisition every 10 s. Processing and analysis of FDAP data was performed as described in [43].

### FRET

PtK2 cells were cotransfected with GFP and mCherry fusion proteins for 24 h as previously described [44]. Cells were then fixed with 4% PFA followed by mounting in mowiol. The FRET unit available at the Leica SP2 confocal setup equipped with 63× NA 1.4 oil objective was used. Briefly, images were first acquired in both green (488 laser) and red (561 laser) channels prior to acceptor (red) photobleaching and were labelled as pre-bleached images. This was followed by bleaching of a 10 µm×10 µm region of interest (ROI) in the acceptor channel (4–8 bleaching cycles using 80% 561 laser power). Following acceptor bleaching, images were acquired in both channels and were labelled as postbleach images. To calculate the FRET efficiency, increase in the fluorescence intensity in the green (donor) channel upon bleaching in the red (acceptor) channel was measured in the ROI and quantified as described before [45].

### Immunoelectron Microscopy

Immunoelectron microscopy was performed as described previously [46]. For electron microscopy analysis, optic nerves were fixed over night with 4% paraformaldehyde and 0.2% glutaraldehyde. The optic nerves were then processed as described [46]. Samples were sectioned using a Leica EM FC6 and were immunolabelled with anti-CNPase (1∶250; Sigma-Aldrich, Munich) antibody followed by gold-labelled secondary antibody (size of gold beads, 10 nm; dilution, 1∶80; AURION, Wageningen, Netherlands). The sections were imaged with a Leo 912AB electron microscope equipped with a CCD camera 2048 X2048 (Proscan, Scheuring, Germany).

### Atomic Force Microscopy

AFM pulling experiments were carried out with a MFP-3D (Asylum Research, Santa Barbara, CA, USA) as described before [21]. The spring constant of the silicon nitride cantilevers (OMCL-TR400PSA-3, Olympus, Japan) was individually calibrated by fitting the power spectrum to a simple harmonic oscillator using the Asylum research built in software routines. Proteins were resuspended in AFM buffer (150 mM NaCl, 5 mM KH2PO4, pH 7.4 titrated with KOH) to a concentration of 0.25 mg/ml. 40 µL was pipetted onto a Mica surface and the AFM cantilever was lowered in the drop, and incubated for 10 min to allow the proteins to bind. Afterwards, sample was rinsed three times with AFM buffer. Force versus separation curves were recorded in liquid at room temperature with a constant speed of 1 µm/s.

### Protein Expression and Purification

H14-TEV-MBP14-Cys and H14-TEV- MBP14 F→S-Cys were expressed from pSF1625 in *E. coli* strain BLR harbouring plasmid pRil. Cultures were grown in TB medium supplemented with 50 µg/ml kanamycin and 37 µg/ml chloramphenicol at 37°C to OD_600_ = 6.0, induced with 1 mM IPTG and further shaken at 37°C for 6 h at 37°C. The protein was purified essentially as described for the Nsp1 FG/FxFG repeat domain [47]. Before cell harvest, 1 mM PMSF (phenylmethylsulfonyl fluoride) and 5 mM EDTA were added directly to the culture. Cells were resuspended in 8.3 M guanidinium-hydrochloride (Gua-HCl) containing 2 mM EDTA and 20 mM DTT and lysed by a single round of freezing and thawing. After centrifugation for 60 min at 38,000 rpm, the cleared lysate was supplemented with 100 mM Tris/HCl (pH 8.5) and 1 mM imidazole and applied to a nickel-chelate column. The column was washed with 7.5 Gua-HCl, 100 mM Tris/HCl (pH 8.5), 1 mM EDTA, 1 mM imidazole followed by a second wash step with 8 M urea, 20 mM Tris/HCl (pH 8.0), 1 mM EDTA, 1 mM imidazole. Bound protein was eluted with the same buffer supplemented with 500 mM imidazole, diluted 1∶3 with water, and applied to a thiopyridine-activated, SH-reactive matrix. The matrix was washed with 6 M Gua-HCl, 20 mM Tris/HCl (pH 8.0), 1 mM EDTA, 1 mM imidazole, and eluted with 6 M Gua-HCl, 20 mM Tris/HCl (pH 7.5), 10 mM DTT, applied to a preparative C18 reverse phase HPLC column, eluted with increasing concentrations of acetonitrile in 0.15% TFA, and lyophilised.

### SLBs

The following mole% of lipid mixtures (mimicking inner leaflet myelin composition) was used at a final concentration of 1 mg/ml, cholesterol∶PE∶PIP2∶PC∶PS∶SM (44%∶27%∶2%∶11.5%∶12.5%∶3%). Lipids were dried in a speed vac and then hydrated in 50 mM HEPES containing 100 mM NaCl at 60°C for 1 h followed by sonication until a clear solution of small unilamellar vesicles (SUVs) was obtained. We used 2% HellmanexIII detergent for the cleaning of the coverslips, followed by hydration via multiple washing steps in MilliQ water. SUVs were then spread onto the cleaned and hydrated glass coverslips. After washing the unbound lipids with 50 mM HEPES, either R3-GFP or MBP was incubated for 40 min followed by washing and addition of GUVs. For the preparation of GUVs, electroformation method was used, which yields unilamellar vesicles with diameter ranging from 5 to 100 µm [48]. The perfusion chamber used for vesicle preparation was equipped with two microscope slides, each coated with indium-tin oxide (ITO), which is electrically conductive and exhibits high light transmission in the visible range. GUVs were grown in the perfusion chamber at high temperature (60°C) in presence of water, as a result of lipid swelling under an AC field [48],[49].

### MBP Pull-Down Assay

Large unilamellar vesicles were prepared via extrusion protocol using mini extruder (Avanti-Polar Lipids) according to the guidelines of the manufacturer. Briefly, lipids resembling inner leaflet composition∶cholesterol∶PE∶PIP2∶PC∶PS∶SM (44%∶27%∶2%∶11.5%∶12.5%∶3%) were mixed together at a final concentration of 1 mg. The lipids were dried in speed-vac followed by addition of 1 ml HEPES buffer and brief sonication (5 min, 30% power, and 60°C) in a bath sonicator to obtain a milky hydrated lipid solution. This solution was then subjected to five freeze-thaw cycles followed by passing through mini-extruder (20 times) using polycarbonate membranes with 100 nm pore size. We added 50 µM of either wild type MBP or F→S mutant to 95 µl of LUV solution and incubated at RT for 30 min. The solutions were ultracentrifuged at 100,000 g (Beckman TLA120.1) immediately. The pellet was resuspended in the same volume as supernatant and the fractions were subjected to SDS-PAGE followed by Western blotting.

### ATR (Attenuated Total Reflection) and Transmission FTIR Spectroscopy

Proteins (MBP wild type and F→S mutant) used for IR measurements were 5× lyophilized from 0.05 M HCl to replace trifluoroacetate counterions at protein backbone against chloride ions [50]. All experiments were carried out in D_2_O containing 120 mM KCl or in ddH_2_O with 20 mM NaOH at a Vertex 70 FTIR (Bruker Optics, Ettlingen, Germany). Spectra were acquired using a MCT detector and a resolution of 2 cm^−1^. All spectra were corrected for water vapour and CO_2_ vibrations. Proteins were measured in solution (4 mg/mL) using a transmission FTIR cell for liquids (AquaSpec, Bruker Optics, Ettlingen, Germany). To monitor the binding of MBP to lipids, a solid SLB was spread on a ZnSe crystal covered with Si mounted to an ATR-FTIR measurement cell (BioATRII, Bruker Optics, Ettlingen, Germany). Therefore, lipid stock solutions in chloroform (*c*_lipid_ = 1–10 mg/mL) were mixed in a test tube, and chloroform was removed to produce a lipid film with the desired composition. These films were then dissolved in D_2_O containing 120 mM KCl at a concentration of 1 mg/mL to obtain multilamellar vesicles that can be transformed into single unilamellar vesicles (SUVs; containing 55.5% PC, 27% PE, 12.5% PS, 3% SM, and 2% PIP2; 1 mg/mL) by sonication (50 W, 0.4 s pulse, 30 min) in a vessel resonator (Sonoplus HD 2070, Bandelin, Berlin, Germany). SSLBs were formed by spreading SUV on the Si surface of the ZnSe crystal at temperatures above phase transition temperature *T*_m_ of used lipids [51]. Secondary structure analyses were carried out using QUANT2 software package provided in the OPUS 6.5 software (Bruker Optics, Ettlingen, Germany). This software provides a library of 43 different proteins and compares it with the measured data to determine the percentage of secondary structure of sample.

### Chemical Cross-Linking Experiments

Cell-based cross-linking protocol was adapted from the procedure described before [52]. Briefly, primary oligodendrocytes were grown on six-well plates (approx. 500,000 per well). At 5 DIV, cell medium was aspirated and the wells were washed once with PBS followed by the addition of 0.1–1 mM of the crosslinker disuccinimidyl glutarate (DSG, Thermo Scientific, GmbH, Germany). Cells were incubated with the cross-linker for 30 min on ice. Cross-linking reaction was quenched by the addition of 1 M Tris-HCl, pH 7.5 at a final concentration of 20 mM (10 min incubation). Thereafter, the wells were washed with PBS. Cells were subsequently scraped and incubated for 10 min in lysis buffer (150 mM NaCl, 1 mM EDTA, 1% Triton-X-100, and 20 mM Tris-HCl, pH 7.5 supplemented with protease inhibitors) and centrifuged at 20,817 g (10 min). Supernatants were subjected to SDS-PAGE followed by Western blot analysis.

### In Vitro Aggregation of Peptides Derived from MBP

MBP peptides at a concentration of 2.5 mM were incubated in 25 mM HEPES (pH 7.5), 150 mM KCl, and 0.5 mM MgCl_2_ for several days at 37°C [53]. A 10 µl aliquot of the sample was placed on an EM grid for 2 min followed by fixation in 1% glutaradehyde for 1 min. Samples were then negatively stained with 2% uranyl acetate and imaged using Leo 912AB electron microscope equipped with a CCD camera 2048 X2048 (Proscan, Scheuring, Germany).

### Expression Plasmids

PLP-GFP, CD9-GFP, CD81-GFP, and GFP-MOG have been described [6]. MOG-GFP was kindly provided by M. Ameloot, Hasselt University, Belgium. A list of the plasmids is shown in Table S1.

## Supporting Information

Figure S1 High ordered assemblies of MBP in primary oligodendrocyte cultures. Chemical cross-linking was performed on 5 DIV primary oligodendrocytes using increasing concentration of disuccinimidyl glutarate (DSG). Cell lysates were analyzed for higher ordered assemblies of MBP using Western blotting.
(TIF)

Figure S2 *Shiverer* rescue with GFP-Tm10-MBP. (A) Representative image of wild-type primary oligodendrocytes expressing GFP-TM-MBP and surface stained with antibodies against GFP. Scale bar, 10 µm. (B) GFP-Tm10-MBP was expressed in 4 DIV MBP-deficient *shiverer* oligodendrocytes and immunostained for CNPase (upper panel). Scale bar, 10 µm. Also note the uniform distribution of CNPase in the membrane sheets of the control 4 DIV *shiverer* cell in the lower panel.
(TIF)

Figure S3 Formation of MBP-domains at the ER-plasma membrane interface in PtK2 cells. (A, B) PtK2 cells expressing GFP-TM-MBP were either surface stained (surface GFP) or permeabilized and then stained (total GFP) with GFP antibodies (red). While surface GFP molecules are excluded from the MBP positive ER-PM domains, a colocalization was observed in permeabilized cells as shown by the intensity profile plots along the marked lines (see the merged images). (C) Co-distribution of MBP domains with the ER marker, ER-Tracker. (D) Morphology of MBP domains upon addition of KKXX ER retention sequence to the C-terminus of GFP-TM-MBP. The domains were co-stained against surface glycoproteins using the lectin Concanavalin A. Scale bar, 10 µm.
(TIF)

Figure S4 Formation of intracellular MBP domains in PtK2 cells is independent of the choice of the transmembrane domain. (A) Representative images of PtK2 cells co-expressing GFP-Tm10 or GFP-Tm10-MBP together with membrane-anchored RFP (mem-RFP), where Tm10 represents the transmembrane domain of Tmem10/Opalin. While expression of GFP-Tm10-MBP results in the formation of ER-PM domains from which mem-RFP is excluded, no domain formation was observed with GFP-Tm10. Scale bar, 10 µm. (B) Quantification of colocalization of mem-RFP with the indicated proteins using Pearson's correlation coefficient. Bars show mean ± SD (*n* = 20 cells, ***p<0.001, *t* test). (C) Representative images of PtK2 cells expressing mem-RFP together with either GFP-PLPTM4-MBP or GFP-PLPTM4, where PLPTM4 represents the fourth transmembrane domain of the proteolipid protein. Scale bar, 10 µm. (D) Quantification of colocalization of mem-RFP with the indicated proteins as in (B). Bars show mean ± SD (*n* = 20 cells, ***p<0.001, *t* test). Note that MBP positive ER-PM domains form independent of the choice of the transmembrane domain.
(TIF)

Figure S5 Exclusion of proteins with large cytosolic domains from MBP-positive patches in PtK2 cells. (A) PtK2 cells were co-transfected with mCherry-TM-MBP and PLP-GFP, CD9-GFP, CD81-GFP, or GFP-MAG. Representative images are shown. Each of these proteins is excluded from the MBP-positive domains as shown by the intensity profile plots on the right side along the marked lines in the merged images. (B) Representative images of PtK2 cells co-expressing mCherry-TM-MBP and MOG-GFP (intracellular GFP) or GFP-MOG (extracellular GFP). Scale bar, 10 µm. Quantification of colocalization indicates that a GFP tag within the cytoplasmic domain prevents localization into the MBP-positive domains. Bars show mean ± SD (*n* = 20 cells, ***p<0.001, *t* test). (C) Serial cytoplasmic truncation mutants of Tmem10 were co-expressed together with GFP-TM-MBP in PtK2 cells. Representative images show cells expressing Tmem10 that lacks the entire cytoplasmic domain (Tm10) or Tmem10 containing 30 amino acids in the cytoplasmic domain (Tm10C30). Scale bar, 10 µm. Quantification of colocalization of the indicated truncation mutants with 10, 20, 30, or 40 amino acids in their cytoplasmic domains with GFP-TM-MBP using Pearson's correlation coefficient. Bars show mean ± SD (*n* = 20 cells, *p<0.05, ANOVA, n.s. indicates no significance).
(TIF)

Figure S6 Reversibility of protein extrusion upon retraction of MBP domains. PtK2 cells co-expressing GFP-TM-MBP and mem-RFP were imaged live. Cells were treated with either (A) 10 µM ionomycin or (B) 100 µM sphingosine, and images were captured every 10 s. Scale bar, 10 µm. Note the reversibility of extrusion as shown by the uniform distribution of mem-RFP (arrows) along the plasma membrane as MBP domains retract (arrow heads) following surface charge redistribution. (C) Time-lapse images of PtK2 cells expressing GFP-TM-MBP and surface stained for glycoproteins using fluorophore-conjugated WGA. Note the fusion of two MBP domains with time (arrows). Furthermore, an island of WGA within the MBP-positive domain is gradually extruded (arrow heads). Scale bar, 10 µm. Time is in seconds.
(TIF)

Figure S7 Colocalization of GFP-TM-MBP F→S with ER-Tracker. Typical image of PtK2 cells expressing GFP-TM-MBP F→S live stained with ER-Tracker. Scale bar, 10 µm.
(TIF)

Figure S8 Hydrophobicity of the phenylalanine residues *per se* is sufficient for establishing the exclusion barrier in PtK2 cells. Representative images of PtK2 cells expressing mCherry-TM fused at the C-terminus to either wild-type MBP or with various MBP mutants, namely F→S, F→A, F→Y, and F→I. The cells were also stained with fluorophore-conjugated concanavalin A (ConA) to visualize surface glycoproteins. While MBP F→S and F→A fail to form the domains, F→Y shows an intermediate phenotype with reduced tendency to form domains. In a striking contrast, F→I mutant forms domains similar to wild-type MBP. Scale bar, 10 µm.
(TIF)

Figure S9 Injection of recombinant AAV2 virus into the corpus callosum of shiverer mice. We injected 1.5 µl (6×10^8^ transducing units/µl) recombinant AAV virus carrying the MBP promoter to express either wild-type MBP or the F→S mutant MBP. The virus was injected into the corpus callosum of *shiverer* mice at P21 and animals were perfused 2 wk later. A representative longitudinal section is shown, with areas of partially compacted myelin in AAV/wild-type-MBP-injected animals as compared to the completely uncompacted myelin in AAV/F→S mutant-MBP injected animals. Quantification of number of wraps is shown as a histogram (only axons with at least two wraps were used for the analysis). Bars show mean ± SD (*n* = 4 with ∼50 axons per animal, *p<0.05, ***p<0.001, *t* test). Scale bar, 1 µm.
(TIF)

Figure S10 Selective interaction with the MBP phase allows protein entry. (A) Representative images of 5 DIV primary oligodendrocytes expressing either wild-type MBP (MBP) or the F→S mutant (MBP F→S), both tagged at the C-terminus with an HA tag, and immunostained for MBP. The expressed proteins were visualized by staining for the HA tags. Quantification of colocalization of the indicated proteins with the total MBP signal was calculated using Pearson's correlation coefficient. Bars represent mean ± SD (*n* = 20 cells, ***p<0.001, *t* test). (B) mCherry-TM-MBP was expressed into PtK2 cells together with either Tm10C50 (Tmem10 harboring 50 amino acid in its cytoplasmic domain) or with Tm10C50-MBP (MBP fused to the C-terminus of Tm10C50). Representative images are shown. Scale bar, 10 µm. Quantification of co-localization of the indicated proteins using Pearson's correlation coefficient. Bars show mean ± SD (*n* = 20 cells, ***p<0.001, *t* test). (C) FKBP-TM-mCherry and GFP-FRB-TM-MBP were co-expressed in PtK2 cells in the absence (control) or presence of 100 nM rapamycin. The rapamycin treatment induces the cross-linking of FRB and FKBP. (A) Representative images showing the distribution of FKBP-TM-mCherry in the control and rapamycin-treated sample. Scale bar, 10 µm. Quantification of colocalization of mCherry with MBP-positive domains in the control versus Rapamycin-treated sample using Pearson's correlation coefficient. Bars show mean ± SD (*n* = 20 cells, **p<0.01, *t* test). (D) Mobility of FKBP-TM-mCherry was monitored outside (control) and inside (rapamycin) the MBP-positive domains by bleaching a squared ROI followed by monitoring the recovery. As a positive control, the mobility of MBP domains was monitored (MBP). Typical recovery curves are presented from three independent experiments. Average recovery curves obtained after photobleaching are shown in the right panel. Bars represent mean ± SEM (*n* = 3 independent experiments, *p<0.05, ANOVA).
(TIF)

Figure S11 The F→S mutant of MBP does not form droplets in basic solution. Representative images of wild-type MBP and MBP F→S (5 mg/mL) dissolved in 20 mM NaOH. Scale bar, 5 µm.
(TIF)

Figure S12 Amyolid dye stainings of wild-type and *shiverer* mice. BTA and K114 staining of P18 MBP-deficient *shiverer* and wild-type mice brain.
(TIF)

Table S1 List of plasmids.
(DOC)

## Abbreviations

AAV: adeno-associated virus
ATR-FTIR: attenuated total reflection fourier transform infrared spectroscopy
CNPase: 2′3′-cyclic nucleotide 3′-phosphodiesterase
ConA: concanvalin A
GUVs: giant unilamellar vesicle
MAG: myelin-associated glycoprotein
MBP: myelin basic protein
SLBs: supported lipid bilayers
Tm10: transmembrane protein 10
WGA: wheat germ agglutinin

## References

[1] Hyman AA, Simons K (2012) Beyond oil and water-phase transitions in cells. Science 337: 1047–1049. 22936764 10.1126/science.1223728

[2] Weber Stephanie C, Brangwynne Clifford P (2012) Getting RNA and protein in phase. Cell 149: 1188–1191. 22682242 10.1016/j.cell.2012.05.022

[3] Brangwynne CP, Eckmann CR, Courson DS, Rybarska A, Hoege C, et al (2009) Germline P granules are liquid droplets that localize by controlled dissolution/condensation. Science 324: 1729–1732. 19460965 10.1126/science.1172046

[4] Kato M, Han Tina W, Xie S, Shi K, Du X, et al (2012) Cell-free formation of RNA granules: low complexity sequence domains form dynamic fibers within hydrogels. Cell 149: 753–767. 22579281 10.1016/j.cell.2012.04.017 PMC6347373

[5] Emery B (2010) Regulation of oligodendrocyte differentiation and myelination. Science 330: 779–782. 21051629 10.1126/science.1190927

[6] Aggarwal S, Yurlova L, Simons M (2011) Central nervous system myelin: structure, synthesis and assembly. Trends Cell Biol 21: 585–593. 21763137 10.1016/j.tcb.2011.06.004

[7] Sherman DL, Brophy PJ (2005) Mechanisms of axon ensheathment and myelin growth. Nat Rev Neurosci 6: 683–690. 16136172 10.1038/nrn1743

[8] Barres BA (2008) The mystery and magic of glia: a perspective on their roles in health and disease. Neuron 60: 430–440. 18995817 10.1016/j.neuron.2008.10.013

[9] Wu LM, Williams A, Delaney A, Sherman DL, Brophy PJ (2012) Increasing internodal distance in myelinated nerves accelerates nerve conduction to a flat maximum. Curr Biol 22: 1957–1961. 23022068 10.1016/j.cub.2012.08.025 PMC3482659

[10] Lee S, Leach MK, Redmond SA, Chong SYC, Mellon SH, et al (2012) A culture system to study oligodendrocyte myelination processes using engineered nanofibers. Nat Methods 9: 917–922. 22796663 10.1038/nmeth.2105 PMC3433633

[11] Poliak S, Peles E (2003) The local differentiation of myelinated axons at nodes of Ranvier. Nat Rev Neurosci 4: 968–980. 14682359 10.1038/nrn1253

[12] Salzer JL, Brophy PJ, Peles E (2008) Molecular domains of myelinated axons in the peripheral nervous system. Glia 56: 1532–1540. 18803321 10.1002/glia.20750

[13] Boggs J (2006) Myelin basic protein: a multifunctional protein. Cellular and Molecular Life Sciences 63: 1945–1961. 16794783 10.1007/s00018-006-6094-7 PMC11136439

[14] Harauz G, Ishiyama N, Hill CMD, Bates IR, Libich DS, et al (2004) Myelin basic protein-diverse conformational states of an intrinsically unstructured protein and its roles in myelin assembly and multiple sclerosis. Micron 35: 503–542. 15219899 10.1016/j.micron.2004.04.005

[15] Harauz G, Ladizhansky V, Boggs JM (2009) Structural polymorphism and multifunctionality of myelin basic protein. Biochemistry 48: 8094–8104. 19642704 10.1021/bi901005f

[16] Roach A, Boylan K, Horvath S, Prusiner SB, Hood LE (1983) Characterization of cloned cDNA representing rat myelin basic protein: absence of expression in brain of shiverer mutant mice. Cell 34: 799–806. 6194889 10.1016/0092-8674(83)90536-6

[17] Aggarwal S, Yurlova L, Snaidero N, Reetz C, Frey S, et al (2011) A size barrier limits protein diffusion at the cell surface to generate lipid-rich myelin-membrane sheets. Dev Cell 21: 445–456. 21885353 10.1016/j.devcel.2011.08.001

[18] Alexander RT, Jaumouille V, Yeung T, Furuya W, Peltekova I, et al (2011) Membrane surface charge dictates the structure and function of the epithelial Na+/H+ exchanger. EMBO J 30: 679–691. 21245831 10.1038/emboj.2010.356 PMC3041952

[19] Yeung T, Gilbert GE, Shi J, Silvius J, Kapus A, et al (2008) Membrane phosphatidylserine regulates surface charge and protein localization. Science 319: 210–213. 18187657 10.1126/science.1152066

[20] Frey S, Richter RP, Görlich D (2006) FG-rich repeats of nuclear pore proteins form a three-dimensional meshwork with hydrogel-like properties. Science 314: 815–817. 17082456 10.1126/science.1132516

[21] Mueller H, Butt H-J, Bamberg E (1999) Force measurements on myelin basic protein adsorbed to mica and lipid bilayer surfaces done with the atomic force microscope. Biophys J 76: 1072–1079. 9916039 10.1016/S0006-3495(99)77272-3 PMC1300057

[22] Chen H, McCarty DM, Bruce AT, Suzuki K (1999) Oligodendrocyte-specific gene expression in mouse brain: use of a myelin-forming cell type-specific promoter in an adeno-associated virus. J Neurosci Res 55: 504–513. 10723060 10.1002/(SICI)1097-4547(19990215)55:4<504::AID-JNR10>3.0.CO;2-0

[23] Inoue T, Heo WD, Grimley JS, Wandless TJ, Meyer T (2005) An inducible translocation strategy to rapidly activate and inhibit small GTPase signaling pathways. Nat Meth 2: 415–418. 10.1038/nmeth763 PMC3579513 15908919

[24] Li P, Banjade S, Cheng H-C, Kim S, Chen B, et al (2012) Phase transitions in the assembly of multivalent signalling proteins. Nature 483: 336–340. 22398450 10.1038/nature10879 PMC3343696

[25] Belli M, Ramazzotti M, Chiti F (2011) Prediction of amyloid aggregation in vivo. EMBO Rep 12: 657–663. 21681200 10.1038/embor.2011.116 PMC3128957

[26] Fowler DM, Koulov AV, Balch WE, Kelly JW (2007) Functional amyloid from bacteria to humans. Trends in Biochemical Sciences 32: 217–224. 17412596 10.1016/j.tibs.2007.03.003

[27] Stankoff B, Freeman L, Aigrot M-S, Chardain A, Dollé F, et al (2011) Imaging central nervous system myelin by positron emission tomography in multiple sclerosis using [methyl-11C]-2-(4′-methylaminophenyl)- 6-hydroxybenzothiazole. Ann Neurol 69: 673–680. 21337603 10.1002/ana.22320

[28] Hyman AA, Brangwynne CP (2011) Beyond stereospecificity: liquids and mesoscale organization of cytoplasm. Dev Cell 21: 14–16. 21763600 10.1016/j.devcel.2011.06.013

[29] Updike DL, Hachey SJ, Kreher J, Strome S (2011) P granules extend the nuclear pore complex environment in the C. elegans germ line. J Cell Biol 192: 939–948. 21402789 10.1083/jcb.201010104 PMC3063144

[30] Uversky VN, Oldfield CJ, Dunker AK (2008) Intrinsically disordered proteins in human diseases: introducing the D2 concept. Annu Rev Biophys 37: 215–246. 18573080 10.1146/annurev.biophys.37.032807.125924

[31] Chiti F, Dobson CM (2006) Protein misfolding, functional amyloid, and human disease. Annu Rev Biochem 75: 333–366. 16756495 10.1146/annurev.biochem.75.101304.123901

[32] Ader C, Frey S, Maas W, Schmidt HB, Görlich D, et al (2010) Amyloid-like interactions within nucleoporin FG hydrogels. Proc Natl Acad Sci U S A 107: 6281–6285. 20304795 10.1073/pnas.0910163107 PMC2852002

[33] Adler-Abramovich L, Vaks L, Carny O, Trudler D, Magno A, et al (2012) Phenylalanine assembly into toxic fibrils suggests amyloid etiology in phenylketonuria. Nat Chem Biol 8: 701–706. 22706200 10.1038/nchembio.1002

[34] Maji SK, Perrin MH, Sawaya MR, Jessberger S, Vadodaria K, et al (2009) Functional amyloids as natural storage of peptide hormones in pituitary secretory granules. Science 325: 328–332. 19541956 10.1126/science.1173155 PMC2865899

[35] White R, Gonsior C, Krämer-Albers E-M, Stöhr N, Hüttelmaier S, et al (2008) Activation of oligodendroglial Fyn kinase enhances translation of mRNAs transported in hnRNP A2-dependent RNA granules. J Cell Biol 181: 579–586. 18490510 10.1083/jcb.200706164 PMC2386098

[36] Laursen LS, Chan CW, ffrench-Constant C (2011) Translation of myelin basic protein mRNA in oligodendrocytes is regulated by integrin activation and hnRNP-K. J Cell Biol 192: 797–811. 21357748 10.1083/jcb.201007014 PMC3051817

[37] Wake H, Lee PR, Fields RD (2011) Control of local protein synthesis and initial events in myelination by action potentials. Science 333: 1647–1651. 21817014 10.1126/science.1206998 PMC3482340

[38] Hülsmann Bastian B, Labokha Aksana A, Görlich D (2012) The permeability of reconstituted nuclear pores provides direct evidence for the selective phase model. Cell 150: 738–751. 22901806 10.1016/j.cell.2012.07.019

[39] Crystal AS, Giasson BI, Crowe A, Kung MP, Zhuang ZP, Trojanowski JQ, Lee VM (2003) A comparison of amyloid fibrillogenesis using the novel fluorescent compound K114. J Neurochem 86: 1359–1368. 12950445 10.1046/j.1471-4159.2003.01949.x

[40] Trajkovic K, Dhaunchak AS, Goncalves JT, Wenzel D, Schneider A, et al (2006) Neuron to glia signaling triggers myelin membrane exocytosis from endosomal storage sites. J Cell Biol 172: 937–948. 16520383 10.1083/jcb.200509022 PMC2063736

[41] Kügler S, Hahnewald R, Garrido M, Reiss J (2007) Long-term rescue of a lethal inherited disease by adeno-associated virus-mediated gene transfer in a mouse model of molybdenum-cofactor deficiency. Am J Hum Genet 80: 291–297. 17236133 10.1086/511281 PMC1785341

[42] Kenworthy AK (2007) Fluorescence recovery after photobleaching studies of lipid rafts. Methods Mol Biol 398: 179–192. 18214381 10.1007/978-1-59745-513-8_13

[43] Serrels A, Timpson P, Canel M, Schwarz JP, Carragher NO, et al (2009) Real-time study of e-cadherin and membrane dynamics in living animals: implications for disease modeling and drug development. Cancer Res 69: 2714–2719. 19318551 10.1158/0008-5472.CAN-08-4308

[44] Fogel AI, Stagi M, Perez de Arce K, Biederer T (2011) Lateral assembly of the immunoglobulin protein SynCAM 1 controls its adhesive function and instructs synapse formation. EMBO J 30: 4728–4738. 21926970 10.1038/emboj.2011.336 PMC3243608

[45] Nawaz S, Kippert A, Saab AS, Werner HB, Lang T, et al (2009) Phosphatidylinositol 4,5-bisphosphate-dependent interaction of myelin basic protein with the plasma membrane in oligodendroglial cells and its rapid perturbation by elevated calcium. J Neurosci 29: 4794–4807. 19369548 10.1523/JNEUROSCI.3955-08.2009 PMC6665343

[46] Werner HB, Kuhlmann K, Shen S, Uecker M, Schardt A, et al (2007) Proteolipid protein is required for transport of sirtuin 2 into CNS myelin. J Neurosci 27: 7717–7730. 17634366 10.1523/JNEUROSCI.1254-07.2007 PMC2676101

[47] Eisele NB, Frey S, Piehler J, Gorlich D, Richter RP (2010) Ultrathin nucleoporin phenylalanine-glycine repeat films and their interaction with nuclear transport receptors. EMBO Rep 11: 366–372. 20379223 10.1038/embor.2010.34 PMC2868541

[48] Kahya N, Scherfeld D, Schwille P (2005) Differential lipid packing abilities and dynamics in giant unilamellar vesicles composed of short-chain saturated glycerol-phospholipids, sphingomyelin and cholesterol. Chem Phys Lipids 135: 169–180. 15869751 10.1016/j.chemphyslip.2005.02.013

[49] Garcia-Saez AJ, Schwille P (2010) Stability of lipid domains. FEBS Lett 584: 1653–1658. 20036662 10.1016/j.febslet.2009.12.036

[50] Andrushchenko VV, Vogel HJ, Prenner EJ (2007) Optimization of the hydrochloric acid concentration used for trifluoroacetate removal from synthetic peptides. J Peptide Sci 13: 37–43. 17031869 10.1002/psc.793

[51] Reimhult E, Höök F, Kasemo B (2002) Temperature dependence of formation of a supported phospholipid bilayer from vesicles on SiO_{2}. Phys Rev E 66: 051905. 10.1103/PhysRevE.66.051905 12513521

[52] Friedrichson T, Kurzchalia TV (1998) Microdomains of GPI-anchored proteins in living cells revealed by crosslinking. Nature 394: 802–805. 9723622 10.1038/29570

[53] Olzscha H, Schermann SM, Woerner AC, Pinkert S, Hecht MH, et al (2011) Amyloid-like aggregates sequester numerous metastable proteins with essential cellular functions. Cell 144: 67–78. 21215370 10.1016/j.cell.2010.11.050

