# Supplementary figures and images for: Myelin Membrane Assembly Is Driven by a Phase Transition of Myelin Basic Proteins Into a Cohesive Protein Meshwork
Source: PLoS Biol. 2013 Jun 4;11(6):e1001577. doi: 10.1371/journal.pbio.1001577 (PMC3676292; doi:10.1371/journal.pbio.1001577)

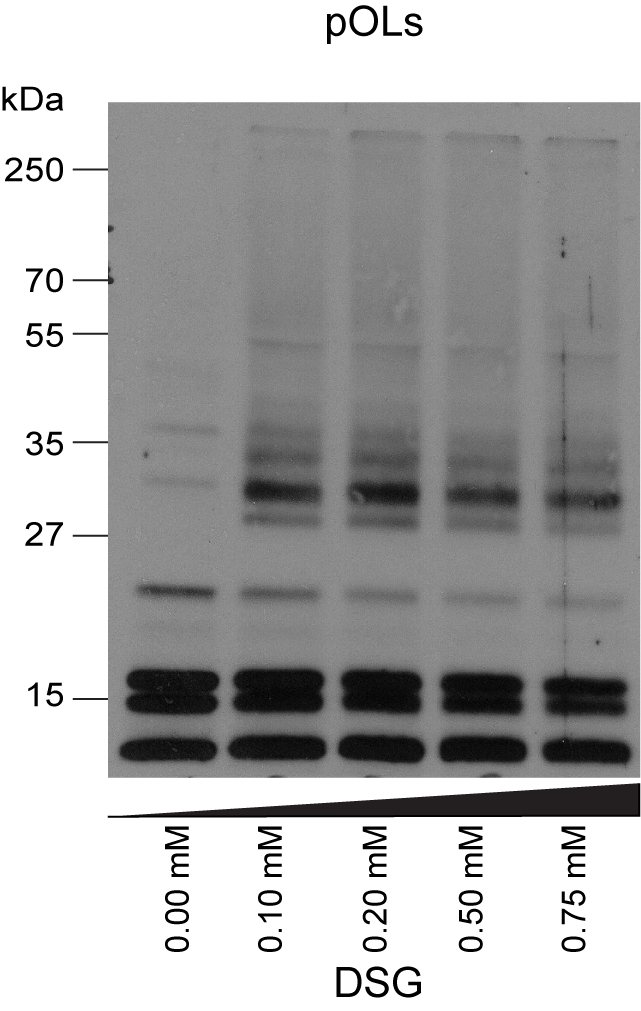

Supplement: Figure S1 — High ordered assemblies of MBP in primary oligodendrocyte cultures. Chemical cross-linking was performed on 5 DIV primary oligodendrocytes using increasing concentration of disuccinimidyl glutarate (DSG). Cell lysates were analyzed for higher ordered assemblies of MBP using Western blotting. (TIF) [file pbio.1001577.s001.tif]

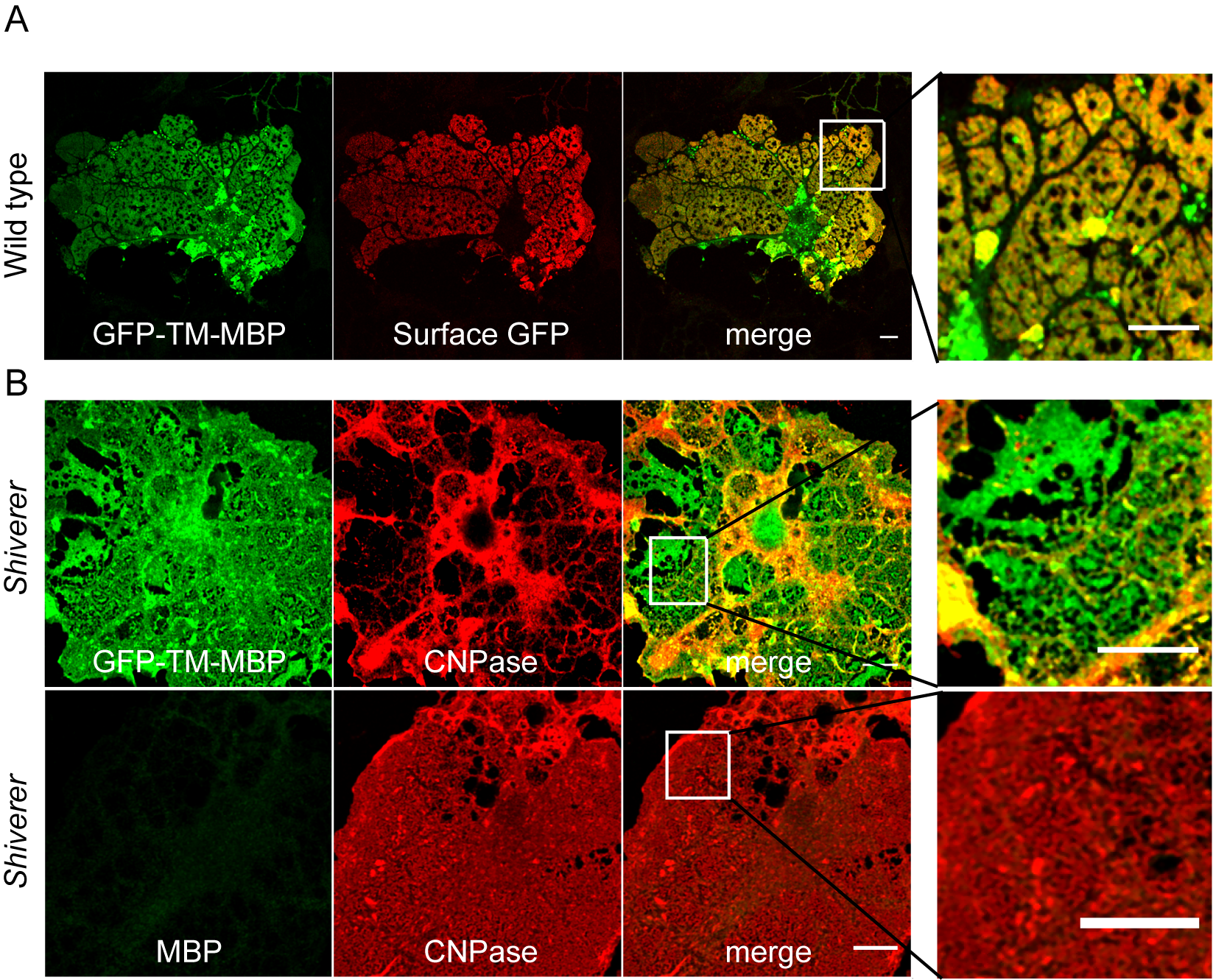

Supplement: Figure S2 — Shiverer rescue with GFP-Tm10-MBP. (A) Representative image of wild-type primary oligodendrocytes expressing GFP-TM-MBP and surface stained with antibodies against GFP. Scale bar, 10 µm. (B) GFP-Tm10-MBP was expressed in 4 DIV MBP-deficient shiverer oligodendrocytes and immunostained for CNPase (upper panel). Scale bar, 10 µm. Also note the uniform distribution of CNPase in the membrane sheets of the control 4 DIV shiverer cell in the lower panel. (TIF) [file pbio.1001577.s002.tif]

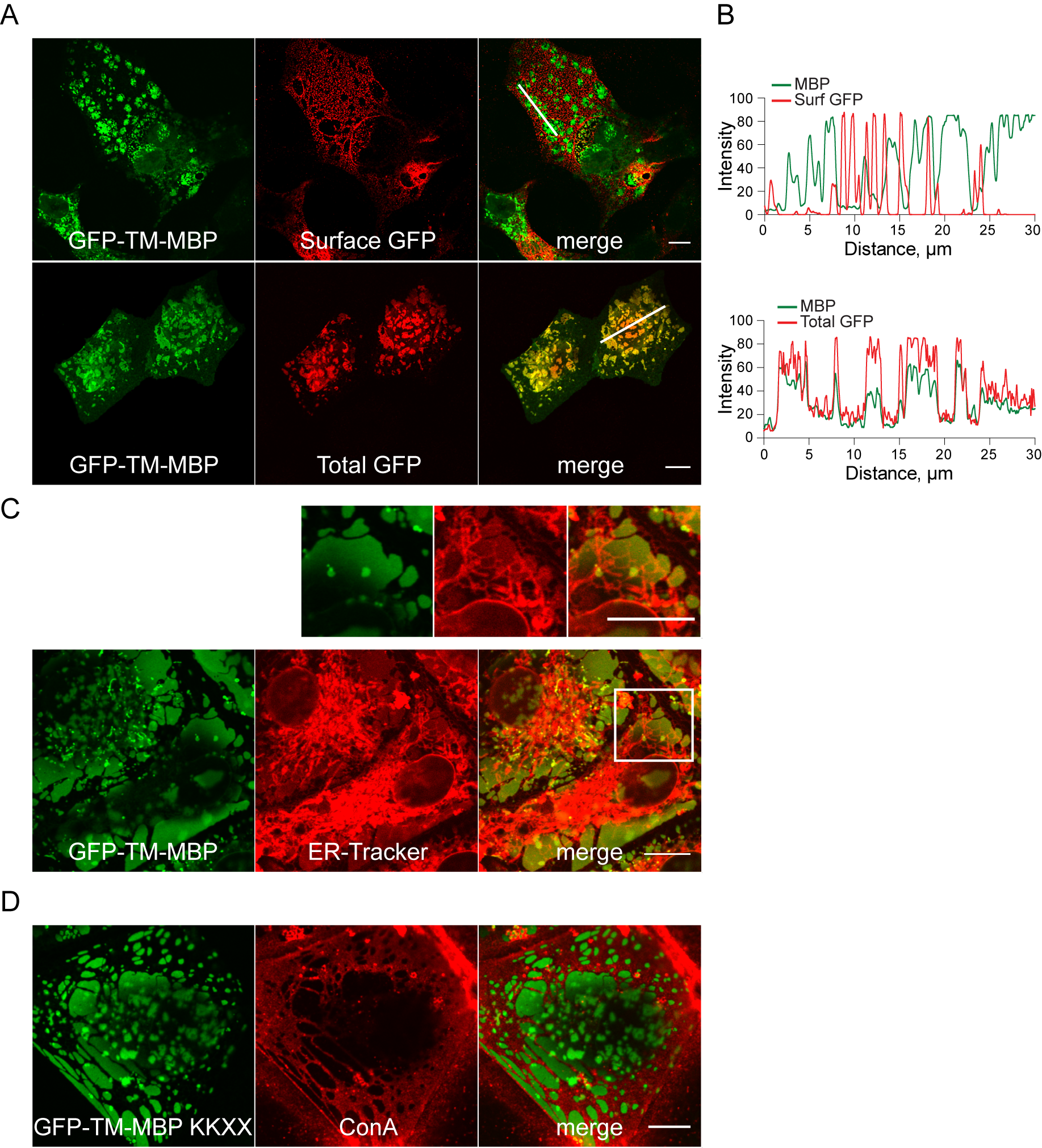

Supplement: Figure S3 — Formation of MBP-domains at the ER-plasma membrane interface in PtK2 cells. (A, B) PtK2 cells expressing GFP-TM-MBP were either surface stained (surface GFP) or permeabilized and then stained (total GFP) with GFP antibodies (red). While surface GFP molecules are excluded from the MBP positive ER-PM domains, a colocalization was observed in permeabilized cells as shown by the intensity profile plots along the marked lines (see the merged images). (C) Co-distribution of MBP domains with the ER marker, ER-Tracker. (D) Morphology of MBP domains upon addition of KKXX ER retention sequence to the C-terminus of GFP-TM-MBP. The domains were co-stained against surface glycoproteins using the lectin Concanavalin A. Scale bar, 10 µm. (TIF) [file pbio.1001577.s003.tif]

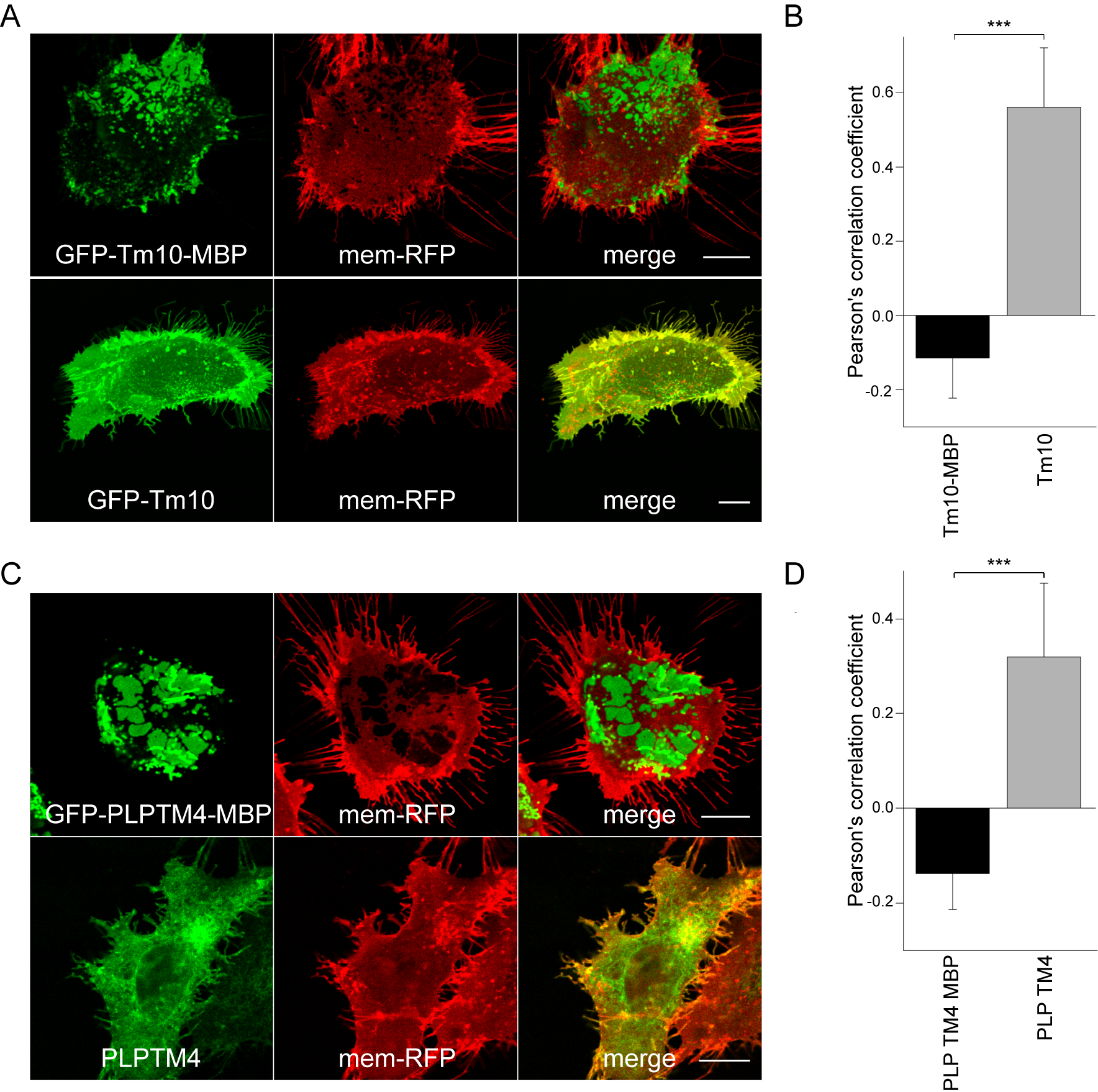

Supplement: Figure S4 — Formation of intracellular MBP domains in PtK2 cells is independent of the choice of the transmembrane domain. (A) Representative images of PtK2 cells co-expressing GFP-Tm10 or GFP-Tm10-MBP together with membrane-anchored RFP (mem-RFP), where Tm10 represents the transmembrane domain of Tmem10/Opalin. While expression of GFP-Tm10-MBP results in the formation of ER-PM domains from which mem-RFP is excluded, no domain formation was observed with GFP-Tm10. Scale bar, 10 µm. (B) Quantification of colocalization of mem-RFP with the indicated proteins using Pearson's correlation coefficient. Bars show mean ± SD (n = 20 cells, ***p<0.001, t test). (C) Representative images of PtK2 cells expressing mem-RFP together with either GFP-PLPTM4-MBP or GFP-PLPTM4, where PLPTM4 represents the fourth transmembrane domain of the proteolipid protein. Scale bar, 10 µm. (D) Quantification of colocalization of mem-RFP with the indicated proteins as in (B). Bars show mean ± SD (n = 20 cells, ***p<0.001, t test). Note that MBP positive ER-PM domains form independent of the choice of the transmembrane domain. (TIF) [file pbio.1001577.s004.tif]

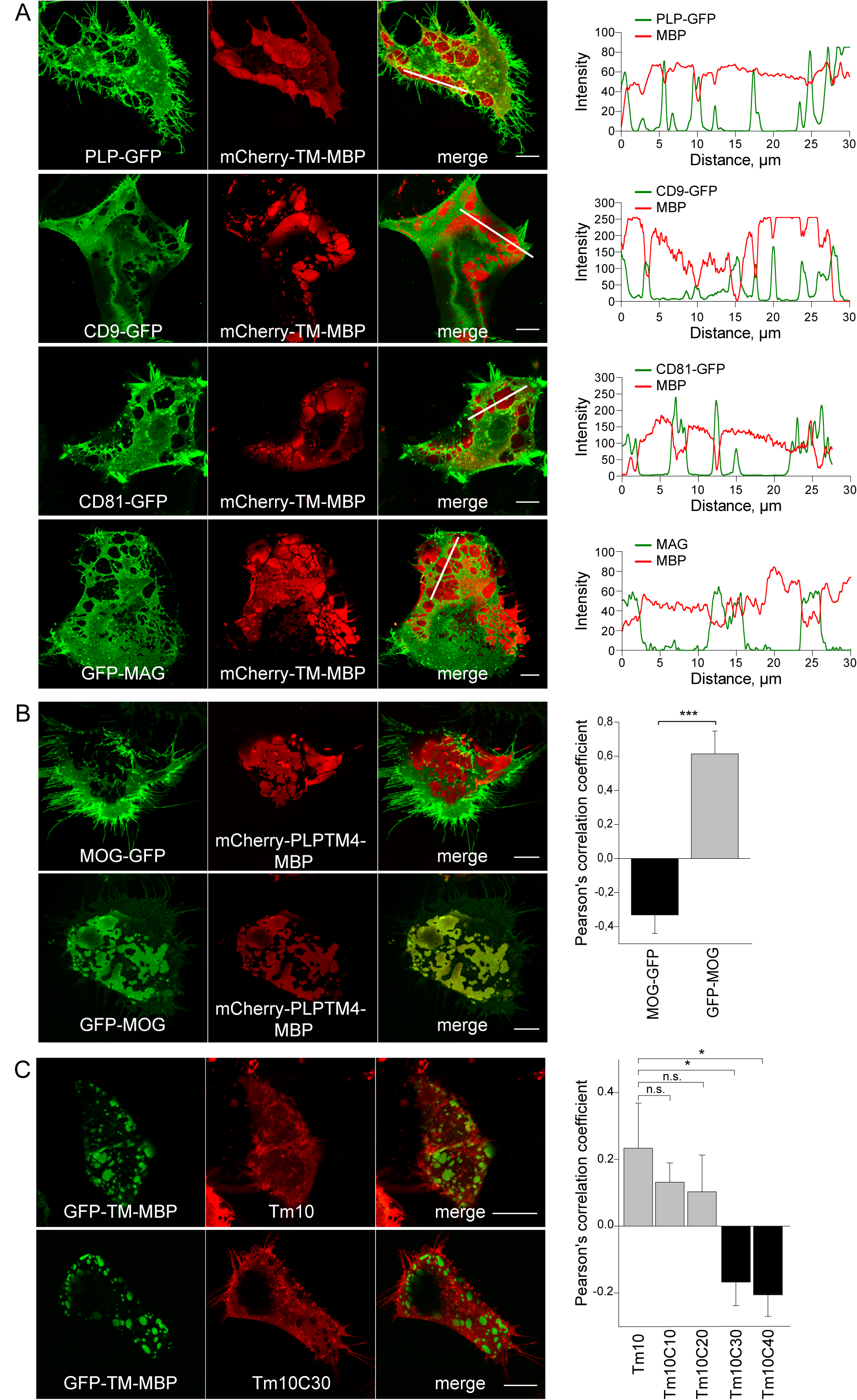

Supplement: Figure S5 — Exclusion of proteins with large cytosolic domains from MBP-positive patches in PtK2 cells. (A) PtK2 cells were co-transfected with mCherry-TM-MBP and PLP-GFP, CD9-GFP, CD81-GFP, or GFP-MAG. Representative images are shown. Each of these proteins is excluded from the MBP-positive domains as shown by the intensity profile plots on the right side along the marked lines in the merged images. (B) Representative images of PtK2 cells co-expressing mCherry-TM-MBP and MOG-GFP (intracellular GFP) or GFP-MOG (extracellular GFP). Scale bar, 10 µm. Quantification of colocalization indicates that a GFP tag within the cytoplasmic domain prevents localization into the MBP-positive domains. Bars show mean ± SD (n = 20 cells, ***p<0.001, t test). (C) Serial cytoplasmic truncation mutants of Tmem10 were co-expressed together with GFP-TM-MBP in PtK2 cells. Representative images show cells expressing Tmem10 that lacks the entire cytoplasmic domain (Tm10) or Tmem10 containing 30 amino acids in the cytoplasmic domain (Tm10C30). Scale bar, 10 µm. Quantification of colocalization of the indicated truncation mutants with 10, 20, 30, or 40 amino acids in their cytoplasmic domains with GFP-TM-MBP using Pearson's correlation coefficient. Bars show mean ± SD (n = 20 cells, *p<0.05, ANOVA, n.s. indicates no significance). (TIF) [file pbio.1001577.s005.tif]

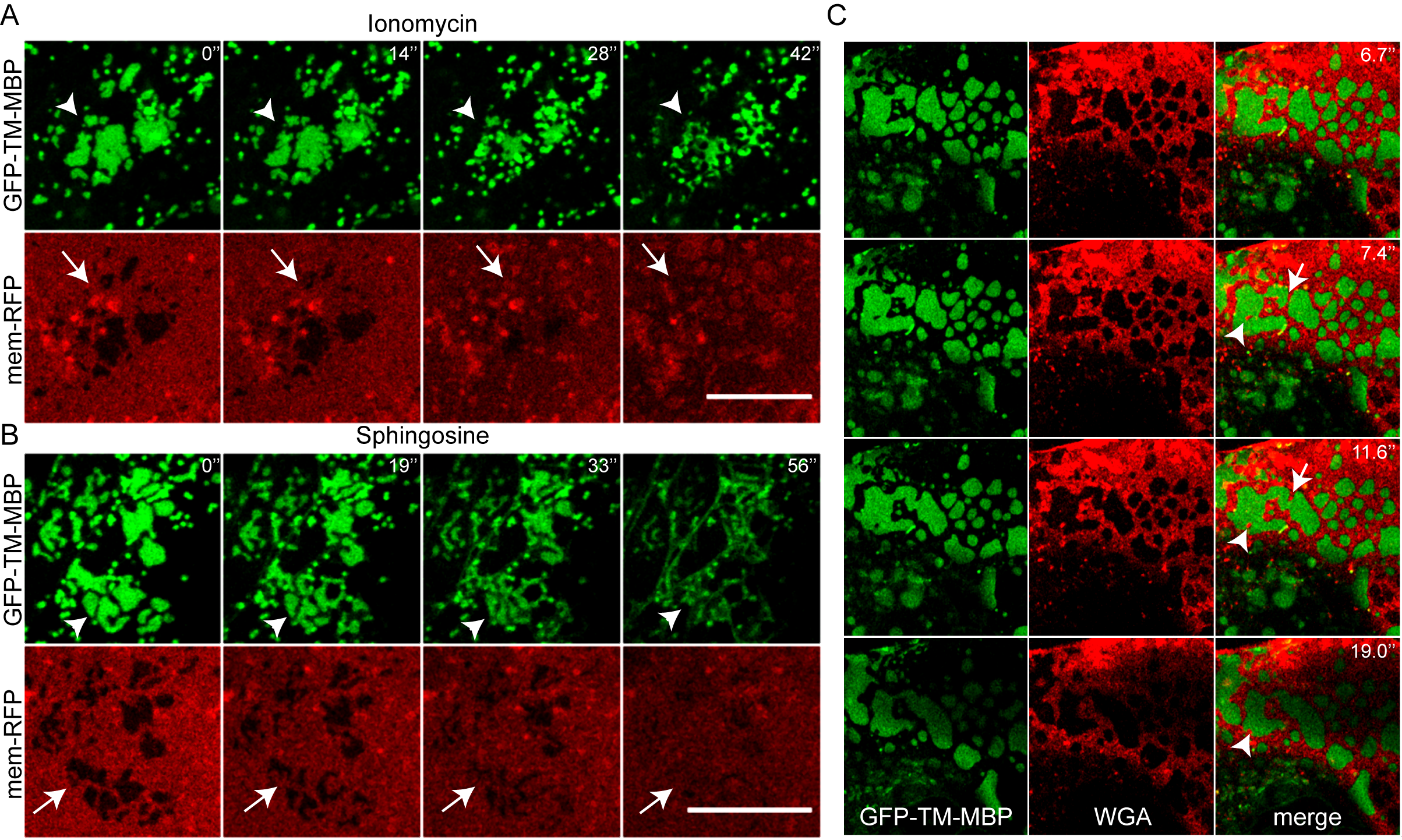

Supplement: Figure S6 — Reversibility of protein extrusion upon retraction of MBP domains. PtK2 cells co-expressing GFP-TM-MBP and mem-RFP were imaged live. Cells were treated with either (A) 10 µM ionomycin or (B) 100 µM sphingosine, and images were captured every 10 s. Scale bar, 10 µm. Note the reversibility of extrusion as shown by the uniform distribution of mem-RFP (arrows) along the plasma membrane as MBP domains retract (arrow heads) following surface charge redistribution. (C) Time-lapse images of PtK2 cells expressing GFP-TM-MBP and surface stained for glycoproteins using fluorophore-conjugated WGA. Note the fusion of two MBP domains with time (arrows). Furthermore, an island of WGA within the MBP-positive domain is gradually extruded (arrow heads). Scale bar, 10 µm. Time is in seconds. (TIF) [file pbio.1001577.s006.tif]

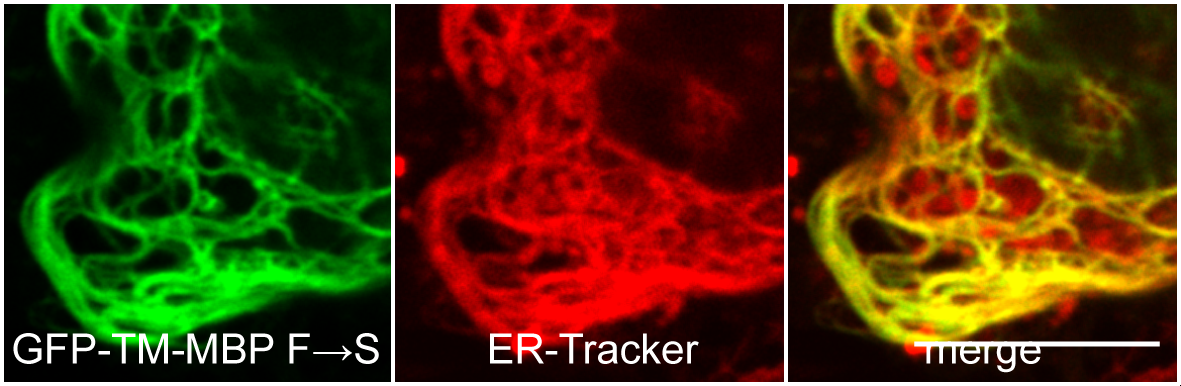

Supplement: Figure S7 — Colocalization of GFP-TM-MBP F→S with ER-Tracker. Typical image of PtK2 cells expressing GFP-TM-MBP F→S live stained with ER-Tracker. Scale bar, 10 µm. (TIF) [file pbio.1001577.s007.tif]

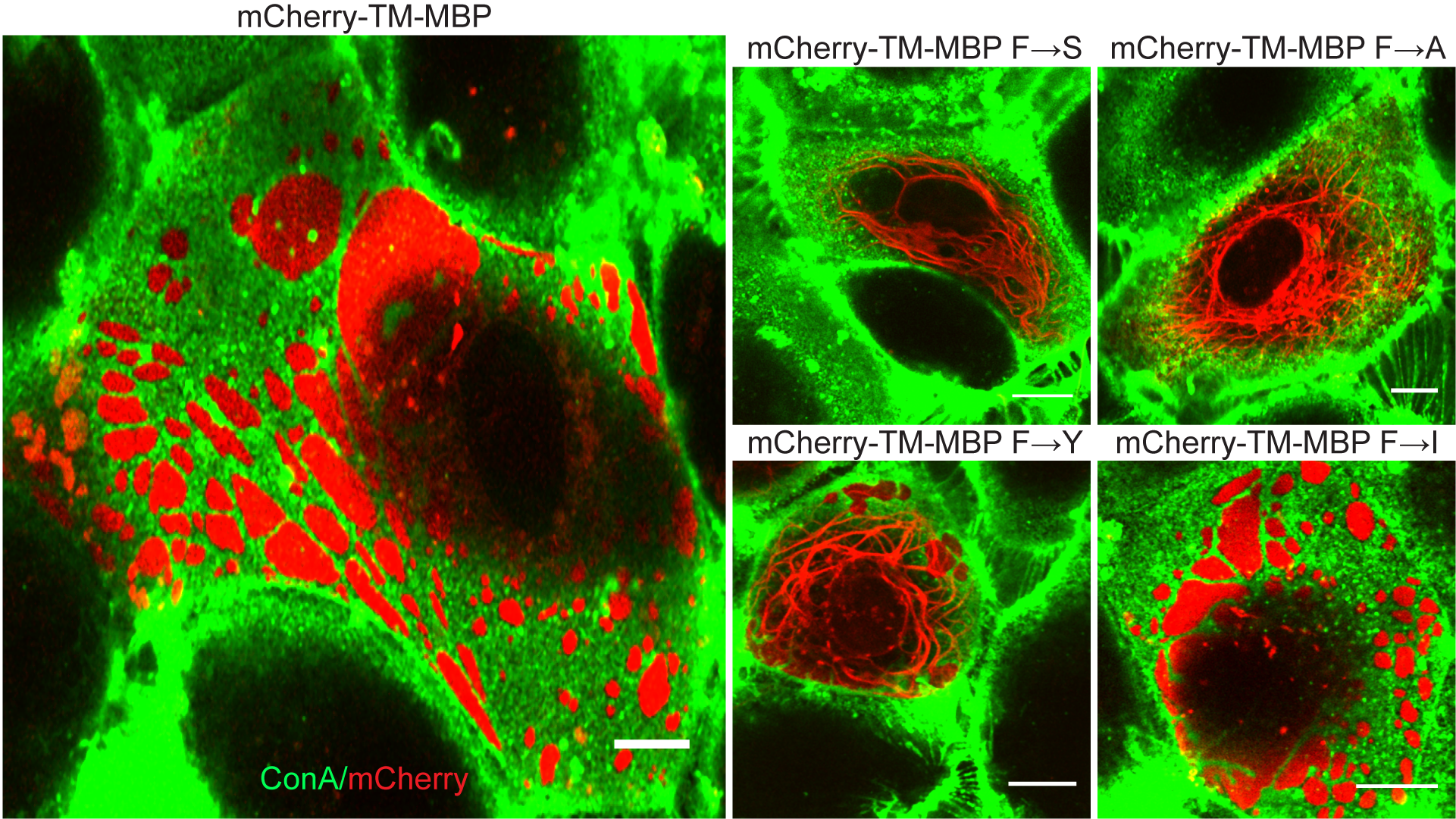

Supplement: Figure S8 — Hydrophobicity of the phenylalanine residues per se is sufficient for establishing the exclusion barrier in PtK2 cells. Representative images of PtK2 cells expressing mCherry-TM fused at the C-terminus to either wild-type MBP or with various MBP mutants, namely F→S, F→A, F→Y, and F→I. The cells were also stained with fluorophore-conjugated concanavalin A (ConA) to visualize surface glycoproteins. While MBP F→S and F→A fail to form the domains, F→Y shows an intermediate phenotype with reduced tendency to form domains. In a striking contrast, F→I mutant forms domains similar to wild-type MBP. Scale bar, 10 µm. (TIF) [file pbio.1001577.s008.tif]

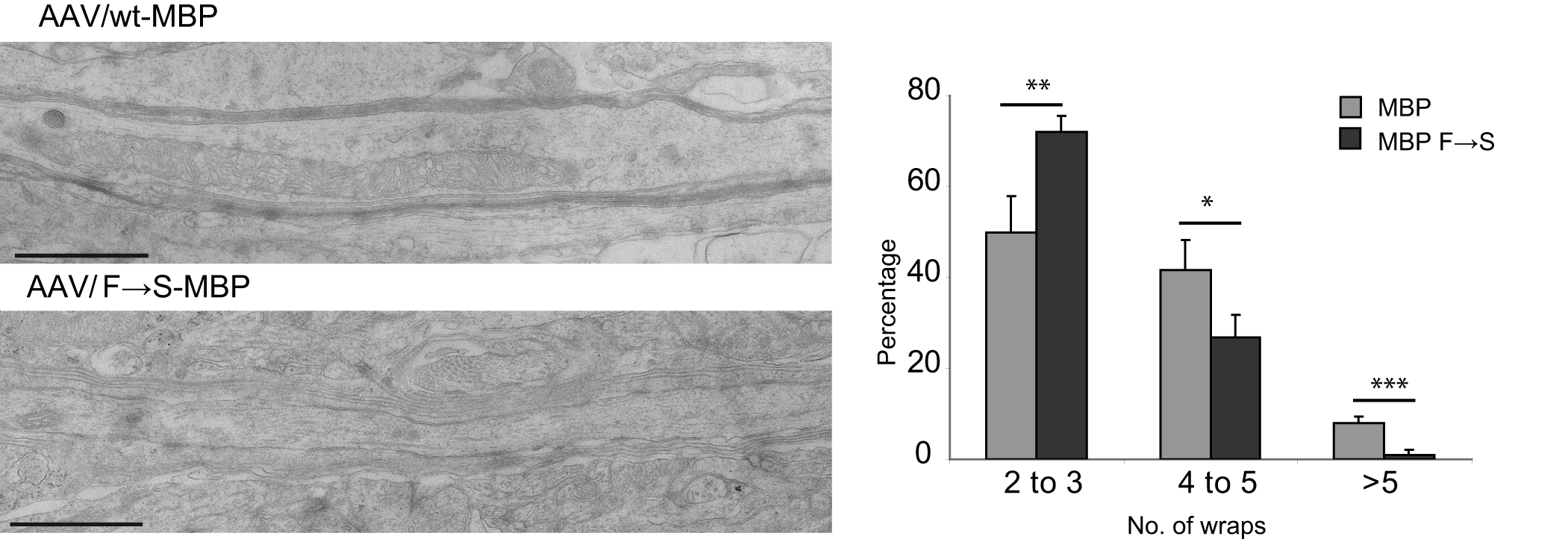

Supplement: Figure S9 — Injection of recombinant AAV2 virus into the corpus callosum of shiverer mice. We injected 1.5 µl (6×10^8^ transducing units/µl) recombinant AAV virus carrying the MBP promoter to express either wild-type MBP or the F→S mutant MBP. The virus was injected into the corpus callosum of shiverer mice at P21 and animals were perfused 2 wk later. A representative longitudinal section is shown, with areas of partially compacted myelin in AAV/wild-type-MBP-injected animals as compared to the completely uncompacted myelin in AAV/F→S mutant-MBP injected animals. Quantification of number of wraps is shown as a histogram (only axons with at least two wraps were used for the analysis). Bars show mean ± SD (n = 4 with ∼50 axons per animal, *p<0.05, ***p<0.001, t test). Scale bar, 1 µm. (TIF) [file pbio.1001577.s009.tif]

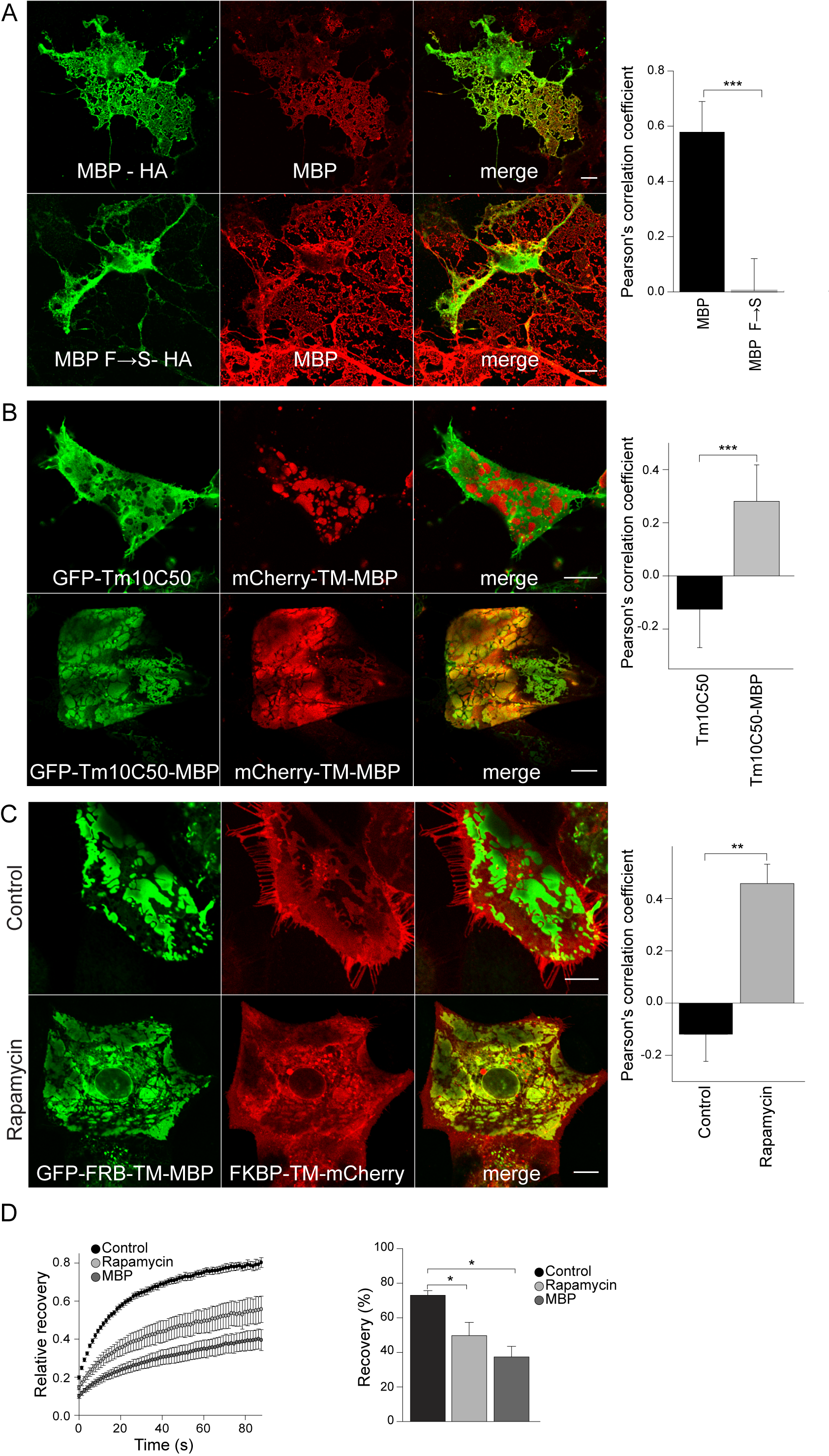

Supplement: Figure S10 — Selective interaction with the MBP phase allows protein entry. (A) Representative images of 5 DIV primary oligodendrocytes expressing either wild-type MBP (MBP) or the F→S mutant (MBP F→S), both tagged at the C-terminus with an HA tag, and immunostained for MBP. The expressed proteins were visualized by staining for the HA tags. Quantification of colocalization of the indicated proteins with the total MBP signal was calculated using Pearson's correlation coefficient. Bars represent mean ± SD (n = 20 cells, ***p<0.001, t test). (B) mCherry-TM-MBP was expressed into PtK2 cells together with either Tm10C50 (Tmem10 harboring 50 amino acid in its cytoplasmic domain) or with Tm10C50-MBP (MBP fused to the C-terminus of Tm10C50). Representative images are shown. Scale bar, 10 µm. Quantification of co-localization of the indicated proteins using Pearson's correlation coefficient. Bars show mean ± SD (n = 20 cells, ***p<0.001, t test). (C) FKBP-TM-mCherry and GFP-FRB-TM-MBP were co-expressed in PtK2 cells in the absence (control) or presence of 100 nM rapamycin. The rapamycin treatment induces the cross-linking of FRB and FKBP. (A) Representative images showing the distribution of FKBP-TM-mCherry in the control and rapamycin-treated sample. Scale bar, 10 µm. Quantification of colocalization of mCherry with MBP-positive domains in the control versus Rapamycin-treated sample using Pearson's correlation coefficient. Bars show mean ± SD (n = 20 cells, **p<0.01, t test). (D) Mobility of FKBP-TM-mCherry was monitored outside (control) and inside (rapamycin) the MBP-positive domains by bleaching a squared ROI followed by monitoring the recovery. As a positive control, the mobility of MBP domains was monitored (MBP). Typical recovery curves are presented from three independent experiments. Average recovery curves obtained after photobleaching are shown in the right panel. Bars represent mean ± SEM (n = 3 independent experiments, *p<0.05, ANOVA). (TIF) [file pbio.1001577.s010.tif]

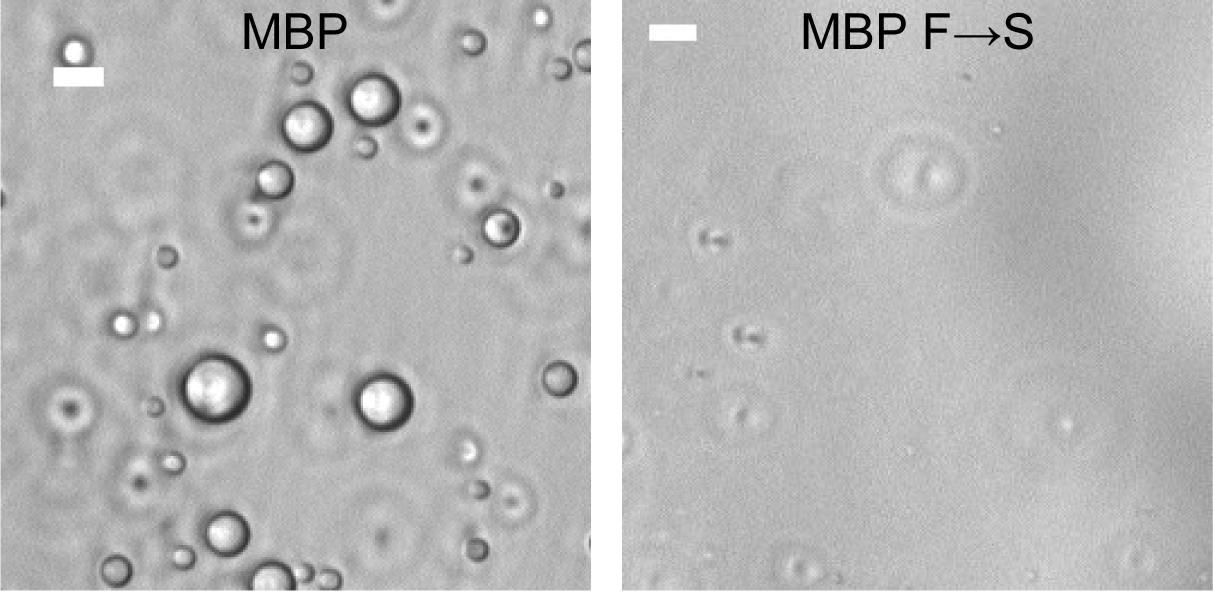

Supplement: Figure S11 — The F→S mutant of MBP does not form droplets in basic solution. Representative images of wild-type MBP and MBP F→S (5 mg/mL) dissolved in 20 mM NaOH. Scale bar, 5 µm. (TIF) [file pbio.1001577.s011.tif]

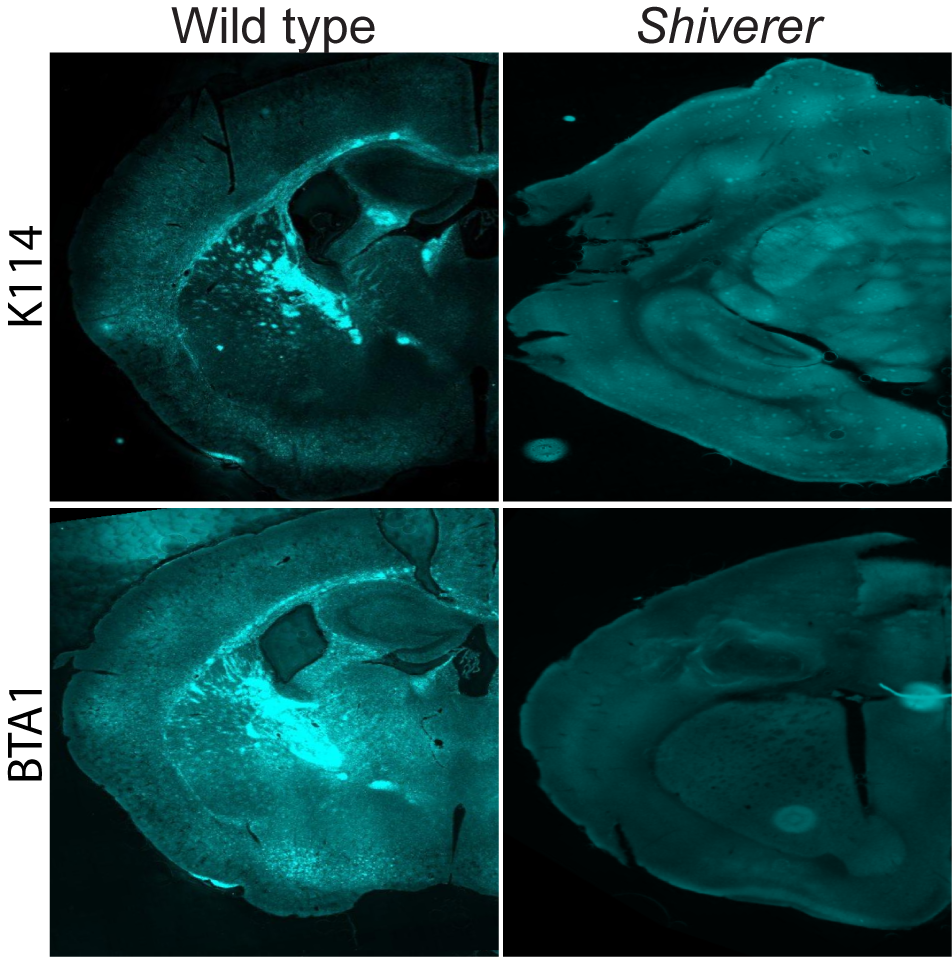

Supplement: Figure S12 — Amyolid dye stainings of wild-type and shiverer mice. BTA and K114 staining of P18 MBP-deficient shiverer and wild-type mice brain. (TIF) [file pbio.1001577.s012.tif]
