# Supplementary material for: Myelin Membrane Assembly Is Driven by a Phase Transition of Myelin Basic Proteins Into a Cohesive Protein Meshwork
Source: PLoS Biol. 2013 Jun 4;11(6):e1001577. doi: 10.1371/journal.pbio.1001577 (PMC3676292; doi:10.1371/journal.pbio.1001577)
Supplement: Table S1 — List of plasmids. (DOC) [file pbio.1001577.s013.doc]

**Table S1. List of constructs used in the study**

| **Plasmids** | **Cloning strategy** | **Vector** | **Resistance** |
| --- | --- | --- | --- |
| SS-myc-mCherry-Tm10-MBP | PCR for mCherry with 25924 and 25925 digested with NotI/AfeI | pcDNA3.1(-) | amp |
| SS-myc-GFP-PLPTM4-MBP | PCR for PLPTM4 with 26184 and26185 digested with AfeI/XhoI | pcDNA3.1(-) | amp |
| SS-myc-GFP-PLPTM4 | PCR for PLPTM4 with 26184 and26185 digested with AfeI/XhoI | pcDNA3.1(-) | amp |
| SS-myc-mCherry-PLPTM4-MBP | PCR for PLPTM4 with 26184 and26185 digested with AfeI/XhoI | pcDNA3.1(-) | amp |
| SS-myc-GFP-MAGNterC46 | PCR for MAGNterC46 with 26318 and 26319 digested with AfeI/HindIII | pcDNA3.1(-) | amp |
| SS-myc-GFP-Tmem10NterC50 | PCR for Tmem10NterC50 with 26320 and 26321digested with AfeI/HindIII | pcDNA3.1(-) | amp |
| SS-myc-mCherry-Tm10Nter | PCR for mCherry with 25924 and 25925 digested with NotI/AfeI | pcDNA3.1(-) | amp |
| SS-myc-mCherry-Tm10NterC10 | PCR for mCherry with 25924 and 25925 digested with NotI/AfeI | pcDNA3.1(-) | amp |
| SS-myc-mCherry-Tm10NterC20 | PCR for mCherry with 25924 and 25925 digested with NotI/AfeI | pcDNA3.1(-) | amp |
| SS-myc-mCherry-Tm10NterC30 | PCR for mCherry with 25924 and 25925 digested with NotI/AfeI | pcDNA3.1(-) | amp |
| SS-myc-mCherry-Tm10NterC40 | PCR for mCherry with 25924 and 25925 digested with NotI/AfeI | pcDNA3.1(-) | amp |
| SS-myc-mCherry-PLPTM4-MBP | PCR for PLPTM4 with 26184 and26185 digested with AfeI/XhoI | pcDNA3.1(-) | amp |
| SS-myc-mCherry-GyPTM | GyPTM by annealing bm01 and bm02 digested with AfeI/XhoI | pcDNA3.1(-) | amp |
| SS-myc-mCherry-GyPTM-MBP | GyPTM by annealing bm01 and bm02 digested with AfeI/XhoI | pcDNA3.1(-) | amp |
| MBP FtoS | ordered from genescript | pUC | amp |
| SS-myc-mCherry-GyPTM-MBP FtoS | PCR for MBP FtoS with 21768 and 21770 digested with BamHI/HindIII | pcDNA3.1(-) | amp |
| SS-myc-GFP-Tm10-MBP FtoS | PCR for MBP FtoS with 22951 and 22952 digested with XhoI/HindIII | pcDNA3.1(-) | amp |
| MBP FtoS-HA | PCR for MBP FtoS with 21768 and 21770 digested with BamHI/HindIII | pcDNA3.1(-) | amp |
| SS-myc-mCherry-PLPTM4-MBP FtoS | PCR for MBP FtoS with 21768 and 21770 digested with BamHI/HindIII | pcDNA3.1(-) | amp |
| His-MBP FtoS | PCR for MBP FtoS with19355 and 19363 digested with BamHI/SpeI | pSFV4 | kan |
| MBP FtoY | ordered from genescript | pUC | amp |
| MBP FtoA | ordered from genescript | pUC | amp |
| MBP FtoI | ordered from genescript | pUC | amp |
| SS-myc-mCherry-GyPTM-MBP FtoY | PCR for MBP FtoY with 21768 and 21770 digested with BamHI/HindIII | pcDNA3.1(-) | amp |
| SS-myc-mCherry-GyPTM-MBP FtoA | PCR for MBP FtoA with 21768 and 21770 digested with BamHI/HindIII | pcDNA3.1(-) | amp |
| SS-myc-mCherry-GyPTM-MBP FtoI | PCR for MBP FtoA with 21768 and 21770 digested with BamHI/HindIII | pcDNA3.1(-) | amp |
| SS-myc-GFP-Tm10-MBP KKXX | MBP 14KD followed by KKXX for ER retention cloned into 195 between XhoI and HindIII | pcDNA3.1(-) | amp |
